# Breast Cancer Organoids Model Patient-Specific Response to Drug Treatment

**DOI:** 10.3390/cancers12123869

**Published:** 2020-12-21

**Authors:** Elena Campaner, Alessandro Zannini, Mariangela Santorsola, Deborah Bonazza, Cristina Bottin, Valeria Cancila, Claudio Tripodo, Marina Bortul, Fabrizio Zanconati, Stefan Schoeftner, Giannino Del Sal

**Affiliations:** 1 Department of Life Sciences, University of Trieste, 34127 Trieste, Italy; ecampaner@units.it (E.C.); azannini@units.it (A.Z.); msantorsola@units.it (M.S.); sschoeftner@units.it (S.S.); 2 National Laboratory CIB (LNCIB), Area Science Park—Padriciano, 34149 Trieste, Italy; 3 Department of Medical and Surgical Sciences, Hospital of Cattinara, University of Trieste, 34149 Trieste, Italy; deborah.bonazza@asugi.sanita.fvg.it (D.B.); cbottin@units.it (C.B.); m.bortul@fmc.units.it (M.B.); fabrizio.zanconati@asugi.sanita.fvg.it (F.Z.); 4 UCO Anatomia e Istologia Patologica, Azienda Sanitaria Universitaria Giuliano Isontina (ASUGI), Hospital of Cattinara, 34149 Trieste, Italy; 5 Tumor Immunology Unit, Department of Health Science, Human Pathology Section, School of Medicine, University of Palermo, 90133 Palermo, Italy; valeria.cancila@unipa.it (V.C.); claudio.tripodo@unipa.it (C.T.); 6 Fondazione Istituto FIRC di Oncologia Molecolare (IFOM), 20139 Milano, Italy; 7 Breast Unit, Division of General Surgery, Azienda Sanitaria Universitaria Giuliano Isontina (ASUGI), Hospital of Cattinara, 34149 Trieste, Italy

**Keywords:** patient-derived tumor organoids, breast cancer, heterogeneity, drug testing, mechanotransduction, YAP, statin, dasatinib

## Abstract

**Simple Summary:**

The possibility to generate in the laboratory faithful models of patients’ tumors is of primary importance to capture cancer complexity and study therapy response in a personalized setting. Tumor organoids are 3D cell cultures, obtained from patients’ tumor tissues, that recapitulate several characteristics of the original tumor, thus representing a clinically relevant patient avatar. This study reports the generation and the molecular characterization of patient-derived organoids from invasive breast carcinomas. Our results proved the usefulness of these cancer models for designing patient-specific therapeutic approaches to treat highly aggressive cancers, but also highlighted the need to further improve this methodology to overcome its current limitations.

**Abstract:**

Tumor organoids are tridimensional cell culture systems that are generated in vitro from surgically resected patients’ tumors. They can be propagated in culture maintaining several features of the tumor of origin, including cellular and genetic heterogeneity, thus representing a promising tool for precision cancer medicine. Here, we established patient-derived tumor organoids (PDOs) from different breast cancer subtypes (luminal A, luminal B, human epidermal growth factor receptor 2 (HER2)-enriched, and triple negative). The established model systems showed histological and genomic concordance with parental tumors. However, in PDOs, the ratio of diverse cell populations was frequently different from that originally observed in parental tumors. We showed that tumor organoids represent a valuable system to test the efficacy of standard therapeutic treatments and to identify drug resistant populations within tumors. We also report that inhibitors of mechanosignaling and of Yes-associated protein 1 (YAP) activation can restore chemosensitivity in drug resistant tumor organoids.

## 1. Introduction

Cancer is a dynamic disease characterized by alterations at the genetic, epigenetic, and transcriptome level, which result in cellular phenotypic changes. Tumor complexity due to the genetic/epigenetic variability between diverse cellular subpopulations, over time and across space, might predispose patients to treatment resistance [1]. This aspect is particularly relevant for breast cancer (BC), where the occurrence of multiple subclones within a tumor mass may lead to ineffectiveness of therapeutic strategies raised against a predominant epithelial clone [2]. As a further level of complexity in this picture, cancer cells are embedded in a tumor microenvironment (TME) composed of stromal cells (e.g., fibroblasts, immune, and endothelial cells) and fibrotic extracellular matrix (ECM). The TME is critical for supporting tumor growth, progression and metastasis, and contributes to the establishment of therapy resistance [2,3,4]. For example, activated fibroblasts can synthesize and crosslink large amounts of collagens, thus creating a fibrotic ECM that reduces the access of cytotoxic drugs within tumors and promotes therapy-resistance via the activation of integrin-mediated mechanotransduction in cancer cells [5].

The generation of patient-specific models that allow a faithful reconstruction of human tumors has primary importance to understand cancer complexity and to develop effective therapeutic treatments. Patient-derived xenografts (PDXs), i.e., engrafts of tumor biopsies into immunodeficient mice, have been developed and used as preclinical models to improve translational research in oncology [6]. Major advantages of these models are represented by the possibility to study the response to drug treatments in vivo, as well as to model metastasis and, to some extent, the TME. However, the use of immunocompromised mice prevents investigating the contribution of the immune system, which is a major limitation of these models [7]. Moreover, maintenance of PDX is time-consuming, expensive, and not suitable for high-throughput screenings. Many of these limitations can be overtaken by the generation of patient-derived tumor organoids (PDOs) [7]. These are tumor cells-derived three-dimensional (3D) cultures generated from cancer biopsies, cultivated in ECM surrogates with specific niche factors. PDOs are able to recapitulate the epithelial architecture of the tumor of origin and to model tumor heterogeneity [8]. This makes them a promising tool to study the response of cancer cells to standard therapy and identify novel therapeutic approaches in a relatively short time. Moreover, PDOs are amenable to long-term maintenance, cryopreservation, and high-throughput drug test [9,10,11].

Recently, in a seminal work, the generation of a biobank of patient-derived breast cancer organoids has been described for its application as a patient-specific model for preclinical studies [10]. In another recent study [12], breast tumor organoids were generated by similar procedures, though these 3D models were described to also contain a significant amount of normal-like cells.

Here, we describe the generation of a collection of patient-derived organoids from invasive breast carcinomas. Molecular and histological characterization of these PDOs, and their usage in drug sensitivity tests, highlighted both advantages and current limitations of these models in cancer research.

## 2. Results

### 2.1. Establishing a Collection of Patient-Derived BC Organoids

**Figure 1:**
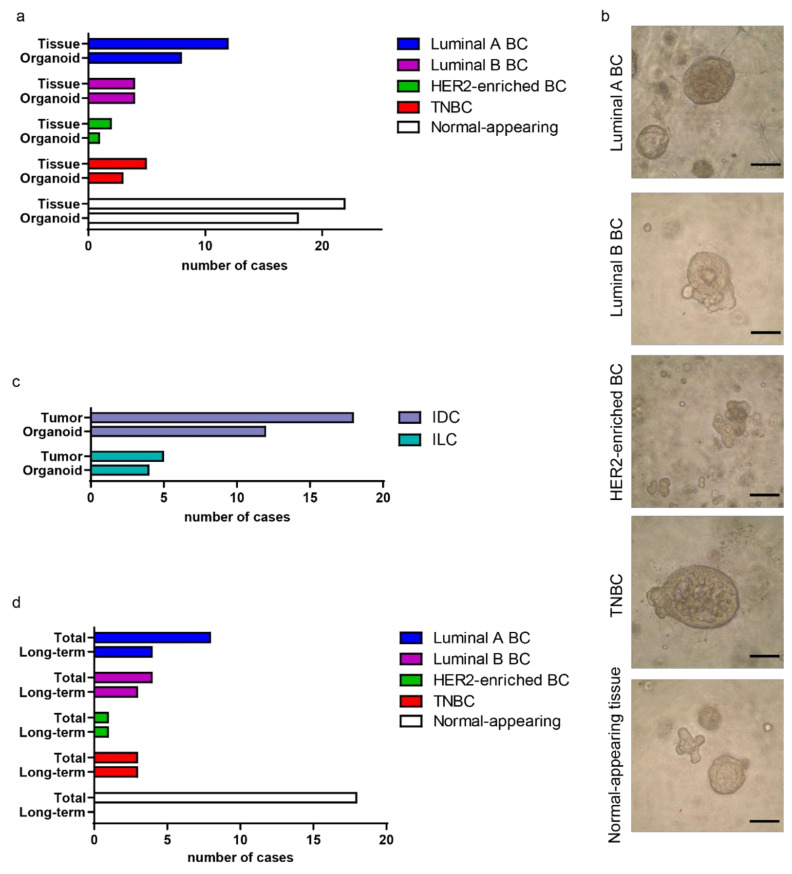
Generating a collection of organoids from primary breast cancers (BCs). (**a**) Histogram representing the stratification of the collected BC, and the derived organoids, based on the molecular subtype. The number of collected normal-appearing adjacent-to-tumor (NAT) specimens and derived organoids is also shown. (**b**) Representative images of organoids derived from BC of different molecular subtypes and from NAT tissue. Scale bar, 100 μm. (**c**) Histogram representing the stratification of the collected BC, and the derived organoids, referring to the histological subtype; IDC, invasive ductal carcinoma; ILC, invasive lobular carcinoma. (**d**) Histogram depicting the number of PDOs maintained in culture for longer time (Long-term) compared with the total number of established cultures, arranged into the molecular subtype. The number of long-term NAT-derived organoids is also shown. Long-term organoids are defined as organoids maintained for more than 4 weeks in culture (passages ≥ 4). TNBC, triple negative BC; HER2, human epidermal growth factor receptor 2.

In order to generate patient-derived BC organoids, 32 patients affected by invasive breast carcinoma were recruited and, immediately after the surgical resection of their treatment-naïve breast tissues, tumor and normal-appearing adjacent-to-tumor (NAT) specimens were collected (Table S1). Epithelial cells were isolated by mechanical dissection of tissues followed by tightly controlled collagenase treatment. After enzymatic treatment, cell clusters were plated in commercially available matrix (Matrigel^®^) to mimic the tumor ECM. Cells were overlaid with an optimized culture medium containing critical compounds and growth factors that allow the generation of BC organoids [10]. As proliferation increased, organoids gradually invaded the matrix and sank into the plate, losing their three-dimensional structure, a process that resulted in the loss of PDOs after a few days of culture (Table S1, cases 1–9). To avoid this phenomenon, previously observed by an independent study [10], starting from case #10, we added a layer of matrix on the bottom of the wells, before plating matrix: cells suspensions, thus creating a barrier to cell sinking. This implementation increased the efficiency of BC organoid generation (cf. organoid generation efficiency before and after case #10 in Table S1). Under these conditions, PDOs were maintained in culture for 5 weeks on average. Viable BC organoids were obtained from 67% of luminal A (8/12), 100% of luminal B (4/4), 50% of human epidermal growth factor receptor 2 (HER2)-enriched (1/2), and 60% of triple negative BC (TNBC; estrogen and progesterone receptors negative, HER2 negative) (3/5) (Figure 1a,b and Table S1). Based on the histological characterization, organoids were generated from both invasive ductal carcinomas (IDCs, 12/18) and invasive lobular carcinomas (ILCs, 4/5) (Figure 1c). The size and morphology of BC organoid cultures varied greatly among different samples, generating solid, cystic, cribriform, and ‘‘grape-like’’ structures.

PDOs’ maintenance in culture over time was higher for more aggressive tumor subtypes, with the lowest proliferation potential for luminal A-derived PDOs and the highest for TNBC and HER2-enriched PDOs (Figure 1d). A clear correlation between time of maintenance of BC organoids in culture and Ki-67 index of the original tumor was observed (Figure S1). Organoids were also obtained from 82% of NAT samples (18/22), with cells arranged to form glandular structures that mimic the mammary ducts (Figure 1a,b). However, NAT-derived organoids proliferated slowly and, after a few passages, the cultures were exhausted (Figure 1d and Table S1).

In sum, we established a collection of 17 patient-derived BC organoids representing the major BC subtypes.

### 2.2. BC Organoids Displayed Histological Features of Parental Tumors

**Figure 2:**
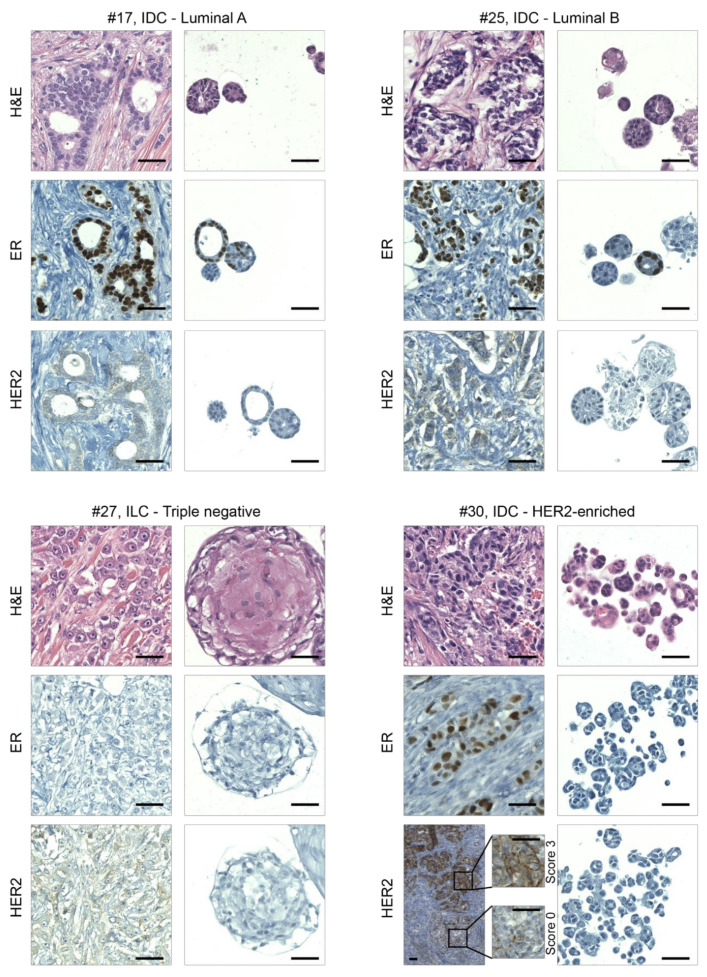
Organoids recapitulate the histological features of primary BCs. Representative images of hematoxylin/eosin staining (H&E) and immunohistochemical analyses on sections of BCs and derived organoids. From left to right and from the top to the bottom are examples of luminal A, luminal B, triple negative, and HER2-enriched BCs, respectively; IDC, invasive ductal carcinoma; ILC, invasive lobular carcinoma; ER, estrogen receptor; HER2, human epidermal growth factor receptor 2. Magnifications of HER2 staining in different areas of cancer tissue #30 are also shown. Scale bar, 100 μm.

BC subtype classification is mainly based on immuno-histological (IHC) analysis of hormone receptors (estrogen and progesterone receptors) and HER2. Hormone receptor positivity has predictive value for the response to endocrine therapy, while HER2 over-expression is determinant for HER2-targeted therapy eligibility and predicts poor prognosis. As a first step in the characterization of the derived organoids, we evaluated the presence of the histological features observed in parental tumors. Although the absence of tumor microenvironmental components in the in vitro models inevitably affects organoids’ morphology, the histological phenotype of BC organoids was similar to that of the cancer cells in the original carcinoma (Figure 2 and Table S1). At the cellular level, PDOs displayed a malignant phenotype characterized by cells with high mitotic activity, apoptosis, and vacuole formation. Differently shaped hyperchromatic nuclei with irregular contours and nuclear molding were also frequently observed (Figure 2 and Figure S2). Analysis of receptor-positive carcinomas and derived organoids revealed that both were predominantly composed of tubular and acinar structures, lined by a single layer of cuboidal cells with scant cytoplasm. TNBC and derived organoids were instead easier to recognize as malignant, characterized by large cells with a solid grown pattern and with prominent macronucleoli (Figure 2 and Figure S2). NAT tissues, instead, generated organoids characterized by a rudimental structure with a hollow lumen surrounded by a monolayer of polarized epithelial cells (Figure S3).

The epithelial and glandular nature of BC-derived organoids was assessed by staining of low-molecular weight keratin (cytokeratin 19), whose expression was found to be similar to that of parental tumors (Figure S4). Regarding hormone receptor analyses, a correlation in the expression of estrogen receptor (ER) between tumor and derived organoids was observed (Figure 2, Table S2); however, in PDOs, hormone receptor expression was reduced compared with primary tumors. We found that HER2 status was maintained in the majority of BC tissue-organoid pairs, as determined by immunohistochemistry (Figure 2). Notably, in PDOs obtained from a HER2-enriched carcinoma (#30), we did not observe HER2 staining. In the parental tumor, only 50% of cancer cells showed high HER2 staining (score 3), while the rest of the tissue had lower or negative staining. This led us to hypothesize that, during organoid expansion, a HER2 negative subclone became dominant over other HER2 positive ones.

In depth analysis of BC tumors and derived PDOs revealed that organoids derived from basal-like tumors retained positivity for the basal marker cytokeratin 5/6 (Figure S5). However, cytokeratin 5/6 positive organoids were also found in cultures derived from luminal cancers. Inspection of parental tumors revealed the presence of cytokeratin 5/6 positive cancer cells within luminal carcinomas as well (Figure S5), a result in line with previous observations [13]. Therefore, it is conceivable that this culture condition might promote the selection of certain basal subclones present at low frequency in the parental tumor.

### 2.3. Genomic Characterization of BC and Derived Organoids

Whole-exome sequencing (WES) was performed in 5 out of 17 patient-derived BC organoids to genomically profile them along with their matched primary tumors and NAT tissue samples (Table S3). Somatic point mutations, somatic copy number variations (SCNVs), and mutational spectra were inferred by WES data and compared among BC tumor-organoid pairs. In this observational study, patients’ blood was not available, thus the NAT tissues were used as healthy control samples to distinguish tumor-specific somatic mutations from patient-specific germline variations. We detected a high number of somatic mutations shared among BC tumor tissues and paired NAT samples (Figure S6a and data not shown), raising concerns, in agreement with previous reports [14,15,16,17], about the use of normal-appearing adjacent-to-tumor tissues as a reference for healthy control tissue.

**Figure 3:**
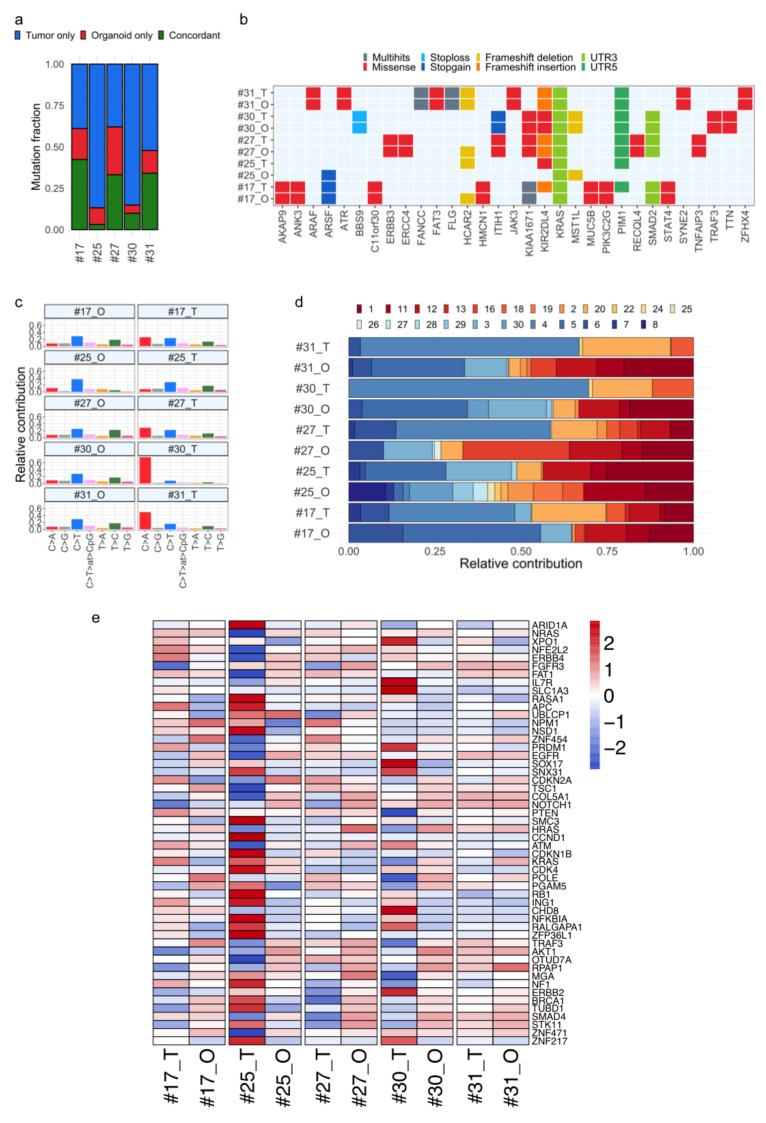
Comparative analysis of mutational signature and cancer genes in BC tumor–organoid pairs. (**a**) Stacked bar chart showing the distribution of somatic mutations specific to tumor tissues (blue) and organoids (red), across five tumor–organoid pairs. The proportion of common mutations between tumor and matched organoid model is indicated in green. (**b**) Overview of somatic nonsynonymous mutations, also affecting cancer genes, shared in tumor and paired organoids. (**c**) Bar graphs representing the relative contribution of the indicated mutation types to the mutational signature extracted by non-negative matrix factorization (NMF) analysis from whole-exome sequencing (WES) data, for each tumor and organoid sample. (**d**) The COSMIC single-base substitution (SBS) signatures (1–30) are conserved among matching tumor–organoid pairs, most of which are annotated as associated to BC (i.e., 1, 2, 3, 5, 13). (**e**) Heat map comparing copy number variations across tumor–organoid pairs in a log2 scale, in BC relevant genes. Red colors indicate gains, and blue colors indicate losses. Samples are labeled as tumor (T) and organoid (O). UTR, untranslated region.

Therefore, in order to identify somatic mutations in tumor and organoid samples, a tumor-only WES analysis workflow was employed, leveraging a virtual panel of normal (PoN) and population variation resources (see Methods). BC tumor tissues showed a greater number of mutations compared with paired organoids, with an average of 4847 and 1602, respectively (Tables S4 and S5). More than 40% of parental tumor mutations were retained in the matched organoids, in three out of five cases (#17, #27, and #31). Less overlap between primary tumors and organoids was observed for cases #25 and #30, involving tumor samples with a relatively high tumor mutational burden (TMB), with more than 7000 total annotated events (Figure 3a and Tables S4 and S5). Candidate pathogenic mutations in untranslated regions (UTRs) and BC-relevant genes regions [18,19], including oncogenes (i.e., *KRAS*, *ERBB3*, *PIK3C2G*) and tumor suppressor genes (i.e., *FANCC*, *ATR*, *SMAD2*), were found in organoids, and the majority of them were conserved within the tumor–organoid pair (Figure 3b, Table S5). In a few cases, mutations in driver genes comprising *ATRX*, *BRCA2*, and *NF1* were found in primary tumors, but not in the derived organoids (Table S5), likely because of the selection in culture of less aberrant cell subpopulations and/or the presence of cell subpopulations at low frequencies in the originating tumors [12].

Next, we interrogated WES data for the presence of BC distinctive mutational signatures [20,21]. Specifically, single-base substitution (SBS) signatures were analyzed and they turned out to be shared within tumor and model pairs (Figure 3c and Figure S6b). Ten out of the 30 COSMIC SBSs relevant to BC were identified in tumor tissues, including kataegis-associated SBS2 and SBS13 (cases #17, #27), aging-related SBS1 and SBS5 (cases #17, #25, and #27), and homologous recombination deficiency-associated SBS3 and SBS8 (cases #17). The majority of them were retained in organoids (Figure 3d).

Finally, a genome-wide SCNV spectra correlation, though at different degrees, was observed among BC tumor-organoid pairs (Spearman’s correlation range 0.39–0.95, Figure S6c,d). SCNVs shared within the tumor-organoid pairs encompassed regions including BC-relevant genes, such as the tumor suppressor genes *APC*, *PTEN*, and *ARID1*; the oncogenes *NRAS,* and *NOTCH1* (Figure 3e). Compared with source tumors, BC organoids #25 and #30 showed different SCNV levels in driver genes such as the oncogenes *ERBB4* (#25), *NRAS* (#25), *ERBB2* (#30), and *XPOI* (#30), and the tumor suppressor genes *APC* (#25), *FAT1* (#25), and *PTEN* (#30). In PDOs #30, *ERBB2* amplification was not observed (Figure 3e), supporting the evidence previously obtained by IHC (HER2 staining, Figure 2).

In summary, patient-derived BC organoids recapitulate, to a different extent, the genome-wide SCNV profile, mutational spectrum, and load observed in patients’ tumor tissues.

### 2.4. BC Organoids as a Platform to Test Drug Sensitivity of Tumors

**Figure 4:**
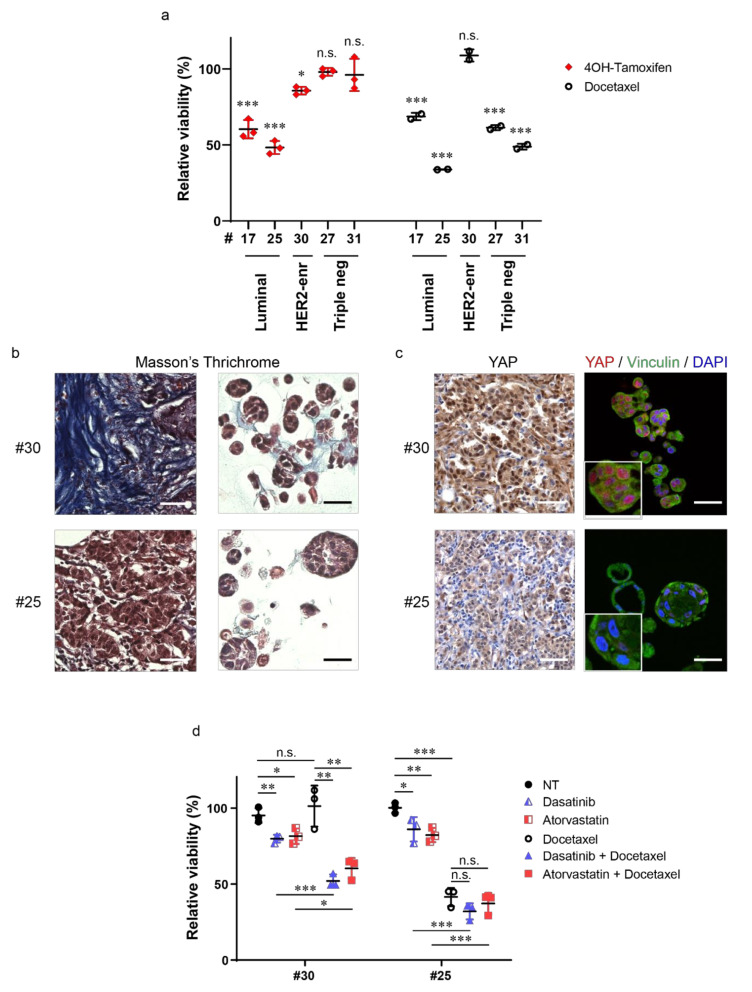
Tumor organoids represent a powerful model to evaluate novel potential therapeutic strategies. (**a**) Dot plot showing relative viability of BC organoids treated with either 10 μM 4-Hydroxytamoxifen (4OH-Tamoxifen) or 1 nM Docetaxel for 7 days. Data are normalized to control treatment (solvent), set to 100%. Ethanol was used as control for 4-Hydroxytamoxifen, while dimethyl sulfoxide (DMSO) was used for Docetaxel. Data shown are the means ± s.d. of *n* = 3 (4OH-Tamoxifen) and *n* = 2 (Docetaxel) independent experiments; * *p* < 0.05, *** *p* < 0.001; n.s., not significant; *t*-test, using the Holm–Sidak method. (**b**) Representative images of Masson’s trichrome staining on sections of BC (left) and derived organoids (right) of the indicated cases. Scale bar, 50 μm. (**c**) Representative images of Yes-associated protein 1 (YAP) immunohistochemistry on BC sections (left) and of YAP (red) and Vinculin (green) immunofluorescence on sections of the derived organoids of the same cases (right). Nuclei were counterstained with either hematoxylin (left) or 4′,6-diamidino-2-phenylindole (DAPI) (right). Scale bar, 50 μm. Magnifications of YAP/Vinculin staining are also shown. (**d**) Dot plot showing relative viability of BC organoids treated with 100 nM Dasatinib, 100 nM Atorvastatin, 1 nM Docetaxel, their combinations, or solvent (NT, DMSO) for 7 days. Data shown are the means ± s.d. of *n* = 3 independent experiments, * *p* < 0.05, ** *p* < 0.01, *** *p* < 0.001; n.s., not significant; *t*-test, using the Holm–Sidak method.

In order to evaluate BC organoids as reliable in vitro disease models, we tested the effect of standard therapies on their viability. To this aim, PDOs #17, #25, #27, #30, and #31 were treated with the ER antagonist Tamoxifen or with the semisynthetic taxane Docetaxel. As shown in Figure 4a, organoids #17, #25, and #30 showed sensitivity to Tamoxifen, while TNBC models were not affected by this treatment. Docetaxel reduced organoids’ viability, with the exception of PDOs #30, which turned out to be resistant to this chemotherapeutic treatment. As described above (Figure 2), sample #30 was originally classified as a heterogenous HER2-enriched tumor, however, the derived organoid model displayed no HER2 amplification and indeed showed resistance to HER2 inhibition (Figure S7).

Emergence of chemoresistance in breast cancer appears to rely on multiple genetic and epigenetic events, as well as on changes in the TME [3,4], including those affecting its physical properties (e.g., increased stiffness due to ECM deposition). Using Masson’s trichrome histochemical staining, which allows evaluation of ECM deposition, we noticed that tumor tissue #30 was characterized by a desmoplastic stroma enriched of an extremely fibrous collagen matrix (Figure 4b). Consistently, we found a prevalent nuclear localization of the mechano-sensitive transcriptional co-activator Yes-associated protein 1 (YAP) [22] in tumor cells, suggesting that ECM alteration increased mechanosignaling in cancer cells (Figure 4c). Notably, tumor organoids derived from this carcinoma also presented collagen staining and nuclear YAP (Figure 4b,c). Increased ECM deposition and YAP activity have been linked to taxane resistance [3,4,22,23]. We thus investigated whether pharmacological inhibition of mechanosignaling and YAP activation might restore sensitivity to Docetaxel in this organoid culture. To this aim, PDOs #30 were treated with Docetaxel in combination with Dasatinib or Atorvastatin. Dasatinib blocks YAP activation by interfering with mechano-transduction through Src inhibition [24], while Atorvastatin, a mevalonate pathway inhibitor, was shown to block YAP activation by reducing RhoA geranyl-geranylation [25]. As shown in Figure 4d, these treatments efficiently restored chemosensitivity of PDOs #30 to Docetaxel. In contrast to case #30, histological analysis of both tumor and derived-organoids of case #25 revealed a lower deposition of collagen and a consistent reduction of nuclear YAP (Figure 4b,c). Accordingly, the sensitivity of PDOs #25 to Docetaxel was not affected by treatment with Dasatinib or Atorvastatin (Figure 4d).

These results indicate that BC organoids could represent a valuable model to test the efficacy of standard therapeutic treatments.

## 3. Discussion

In this work, we established 17 lines of patient-derived breast cancer organoids of different BC subtypes and, for a subset of them, we described genomic features and sensitivity to drug treatment.

The histological characteristics of developed PDOs were concordant with those detected in parental tumors. Moreover, the propagation potential of these PDOs’ lines appeared heterogeneous and resembled the proliferation rate of parental tumors. As already reported for luminal carcinomas [10], expression of estrogen receptor in some BC organoids was reduced compared with primary tumors. In addition, in PDOs, we observed an enrichment of basal cancer cells (cytokeratin 5/6 positive), which have also been detected in parental tumors. The presence of cytokeratin 5/6 positive cells has been already reported for luminal carcinomas [13]. This fact raises the possibility that the in vitro culture might promote the selection of basal subclones that are under-represented in the parental tumor. The enrichment of tumor sub-clones in PDOs may result from a selective pressure owing to the lack of support by cellular and non-cellular components of the TME [26]. Moreover, culture conditions may also differently impact the growth of specific cellular subpopulations. For instance, it is known that, in luminal breast cancer models, upon withdrawal of estrogens, rare ER−/CK5+ cells are enriched [27]. Notably, breast cancer organoids medium does not contain estrogens. Therefore, it is conceivable that the medium recipe we used to culture organoids might negatively affect the proliferation of ER+ cells, thus contributing to ER−/CK5+ cancer cells’ expansion in vitro. Moreover, this medium contains a cocktail of growth factors/ligands known to promote epithelial to mesenchymal transition [28]. Accordingly, Rosenbluth et al. [29], have demonstrated that specific medium components (i.e., EGF, Noggin) affect the relative proportion of mammary lineages that are present in organoid cultures, unfavoring the proliferation of mature luminal cells. Based on this evidence, a precise definition of BC organoids media components is required to better preserve parental tumor features. Developing organoids together with native or reconstituted stromal components might also be a strategy to supply the organoids with specific tumor niche factors [30].

The genetic concordance between tumors and organoids varied among analyzed samples. Indeed, despite an overall consistency of the somatic mutational spectra and copy number profiles across tumor–organoid pairs, less concordance in the overall mutational load, and in some key driver genes, was observed for some cases. In three out of five cases (#17, #27, and #31), more than 40% of point mutations detected in the parental tumors were retained in organoids’ cultures. In cases #25 and #30, with native tumor tissues displaying a higher tumor mutational burden, a weaker correspondence between tumors and derived organoids was observed, likely ascribable to the procedures of tumor tissue sampling and organoid culture development. Along with this, intrinsic tumor features such as purity, stage, and regional heterogeneity [31,32] might have affected the outgrowth efficiency of organoids, causing the loss of certain tumor sub-clones and contributing to the generation of poorly matching tumor–organoid pairs. In light of this result, the generation of BC organoids from different regions of the same tumor would provide a better in vitro representation of intra-tumor heterogeneity [33]. Moreover, not only the establishment of PDOs from primary tumors, but also from metastatic axillary lymph nodes and distant organ metastases of the same patient, might represent an invaluable resource to study disease progression and test sensitivity to drug treatments [10,34]. The latter application is particularly relevant for metastases. Indeed, the therapeutic strategies used for the metastatic disease are often the same as those used for treating the primary tumor [35], and therapy resistance is frequently observed. In this context, the generation of organoids from BC metastases could provide valuable models to study metastasis dependencies and test specific lines of interventions. However, only highly proliferating organoids are amenable to drug screening, and this represent a weakness of the model that should be implemented.

PDOs can also represent platforms to test combination treatments aimed at finding drug synergisms to overcome therapy resistance and provide second lines of intervention [36]. In our study, treatment of PDOs with conventional therapies allowed us to identify both potential responders and non-responders. An example was provided by case #30. PDOs #30 were obtained from a tumor classified as HER2-enriched, but IHC and WES analyses of these PDOs revealed that the derived organoid line was HER2 negative, raising the possibility that it originated from a HER2 negative cancer cell subpopulation of the parental tumor. This organoid line was employed as a model of a tumor that becomes refractory to anti-HER2 treatment, thus it was used to identify alternative therapeutic approaches. Interestingly, and similarly to the tumor of origin, PDOs #30 exhibited collagen fiber deposition and consequently increased nuclear levels of the mechanotransducer YAP. Altered ECM deposition and high levels of YAP are reported to confer resistance to chemotherapy [3,4,22,23]. Of note, PDOs #30 were insensitive to taxane treatment, while combined treatment with mechanosignaling/YAP inhibitors [37] rescued their sensitivity to the drug. With regard to the observed effect of the combined treatment, inhibition of mechanotransduction has already been reported to improve anti-cancer therapy efficacy in melanoma and in pancreatic cancer [38,39]. The hypothesis of combining chemotherapy with mechanosignaling inhibitors to overcome BC drug resistance is worth further investigating in other PDOs derived from desmoplastic BCs and in mouse models of BC. A few clinical trials have already been testing such combinations in BC and their results will provide indications on the efficacy of this therapeutic approach (NCT03358017, NCT00820170).

## 4. Materials and Methods

### 4.1. Bioethics Approval of Studies Involving Humans and Patient Informed Consent

The protocol n.62/2017 for the establishment of mammary gland organoid cultures as well as their biobanking (title of the observational study: ‘*Sviluppo di metodologie e protocolli per l’isolamento di organoidi da campioni umani di tumore del polmone, mesotelio, mammella*’) has received approval from the bioethical committee of the Friuli Venezia Giulia region of Italy (C.E.U.R.—FVG, approval n.233 REC.DEC 28/03/2018). The protocol comprises policy rules for human biological samples and related data ensuring that the biological material has been exclusively obtained from approved sources and has an owner that agrees with the acquisition and specific use of the biological sample by signing an informed consent document. Biological samples are traceable and are uniquely identifiable by a coding system that protects the donors’ identity from experimenters that perform research. The informed patient consent document allows the use of the biological material for non-profit research, allows the withdrawal of consent at any time, regulates use of genomics data, and specifies the period of time the biological material can be retained. All patients participating in the observational study obtained detailed information in an oral conversation with the treating physician and signed informed consent forms. Patients affected by non-metastatic treatment-naïve invasive breast carcinoma were recruited at the Cattinara hospital of Trieste in the Friuli Venezia Giulia region of Italy.

### 4.2. Breast Tissue Selection and Processing

After the arrival of breast specimens to the hospital’s pathology unit, relevant regions of tumor tissue and normal-appearing adjacent-to-tumor tissue for routine diagnosis and for research purpose were selected. Scrape cytology of cell material brushed from tumor tissue cross section revealed information on tissue quality, abundance, and morphology of tumor cells. The size of tumor tissue dedicated to organoids generation was in the range of 1–2 cm^3^ and it was contiguous to the diagnostic tissue. Resected normal-appearing adjacent-to-tumor tissue was obtained from tumor-free surgical margins and its morphology was controlled by pathologists.

### 4.3. BC Organoid Culture

For organoid generation, tissues were processed as detailed in Supplementary Methods. Briefly, samples were finely minced, a part was snap frozen and stored at −80 °C as back-up, and the remaining tissue was processed for the isolation of viable cells. Enzymatic digestion of minced tissues with collagenase A (1.6 U/mL) for 1–2 h at 37 °C was followed by elimination of red blood cells using ammonium chloride solution. Isolated cell clusters were resuspended in growth factor reduced solubilized basement membrane (Matrigel^®^, Corning) and plated into drops in 24-well plates. BC organoid medium [10] was added to each well and changed every 3–4 days. Breast organoid medium consists of Advanced DMEM:F12 (Gibco) supplemented with 1X Glutamax, 10 mM Hepes, 1X Penicillin/Streptomycin, 50 µg/mL Primocin, 1X B27 supplement, 5 mM Nicotinamide, 1.25 mM N-Acetylcystein, 250 ng/mL R-spondin 3, 5 nM Heregulin β-1, 100 ng/mL Noggin, 20 ng/mL FGF-10, 5 ng/mL FGF-7, 5 ng/mL EGF, 500 nM A83-01, and 500 nM SB202190. Moreover, 5 μM Y-27632 was added to culture media for the first three days of culture. Organoids were passaged when confluency was reached, usually once a week. For organoids’ passaging, Matrigel was displaced from the wells and collected. The material was incubated for 1 h at 4 °C with Cell Recovery solution (Corning), which allows Matrigel solubilization and organoids’ release. Organoids were then digested with TrypLE solution (Gibco) for 5 min at 37 °C. After enzyme neutralization and washing, organoid fragments were resuspended in Matrigel and reseeded as above in order to allow the formation of new organoids. Bright-field imaging of organoids was performed on an Olympus CK30 microscope.

### 4.4. Histology of Tissues and Organoids

Tissues dedicated to routine diagnosis were fixed in 10% buffered formalin. After dehydration, they were paraffin embedded and sections were cut to a thickness of 3 μm. Organoids were released from Matrigel and fixed in 4% buffered paraformaldehyde, washed in phosphate-buffered saline (PBS), and resuspended in pre-dissolved 1% agarose. Agarose blocks were embedded in paraffin and sectioned as human tissues. For standard H&E staining, slides were deparaffinized in xylene, rehydrated by graded alcohol, and stained with hematoxylin and eosin to appreciate the cellular and tissue structure details. Immunohistochemical stainings were performed on sections 3 μm thin in the automated Benchmark Ultra platform (Ventana Medical System Inc., Tucson, AZ, USA), using prediluited Ventana Primary Antibodies and Ventana UltraView Universal DAB detection kit (Roche Diagnostics). Counterstaining and post-counterstaining were performed using hematoxylin and bluing reagent (Ventana, Roche Diagnostics). For molecular subtype determination of breast carcinomas, ER, PR, HER2, and Ki-67 have been scored according to the last version of the American Society of Clinical Oncology/College of American Pathologists (ASCO/CAP) guidelines [40,41,42]. Histochemistry was performed using Artisan Masson’s trichrome Stain Kit in Dako Autostainer. Images were acquired on a D-sight brightfield slide scanner (Menarini Diagnostics) and analyzed by breast cancer pathologists. For immunohistochemistry of YAP, antigen retrieval was performed utilizing the Novocastra Epitope Retrival Solution pH9 (Novocastra, Leica Biosystems) at 98 °C for 30 min. After neutralization of the endogenous peroxidase with 3% H_2_O_2_, Fc blocking by Novocastra Protein Block (0.4% Casein in phosphate-buffered saline) was performed, and then samples were incubated with mouse monoclonal YAP antibody (Santa Cruz). Staining was revealed using a polymer detection kit (Novocastra, Leica Biosystems) and DAB as chromogenic substrate. Negative control for YAP immunostaining is shown in Figure S8. Slides were analyzed under an Axioscope A1 microscope equipped with Axiocam 503 Color camera (Zeiss). Antibody specifications are listed in Table S6.

### 4.5. Immunofluorescence of Organoids

For immunofluorescence analysis, organoids were processed as above. Organoids sections were deparaffinized, rehydrated, and antigen retrieved in a steam pressure cooker with citrate buffer (pH 6.0). The slides were blocked in 0.1% bovine serum albumin (BSA), 0.2% Triton X-100, and 0.05% Tween 20 in PBS for 1 h at room temperature (RT). The slides were then incubated overnight with antibodies against YAP (Abcam) and Vinculin (Sigma) in blocking buffer at 4 °C. After washing, slides were incubated with goat anti-rabbit Alexa Fluor 568 and goat anti-mouse Alexa Fluor 488 (Life Technologies) for 45 min at 37 °C. Nuclei were counterstained with DAPI (Sigma). Images were acquired on a Nikon Eclipse C1si confocal microscope and processed using the Fiji software package.

### 4.6. Genomic Analysis

#### 4.6.1. Whole Exome Sequencing and Read Alignment

Genomic DNA isolation from diagnostic tissues (formalin-fixed paraffin-embedded tissues, FFPE) was performed by Macrogen (Rep. of Korea) using the Maxwell^®^ 16 FFPE Tissue LEV DNA Purification Kit (Promega). gDNA was isolated from organoids using the QIAamp DNA Mini Kit (QIAGEN). DNA libraries were generated using Agilent SureSelect Human All Exon V6 (Agilent technology) protocol and sequenced on Illumina NovaSeq platform, with paired-end (2 × 150bp) runs. Sequencing depth had a mean of 78 and 119×, with 92 and 98% of coverage mean in the target region, in tumor and organoid samples, respectively (Table S2).

To confirm tumor and NAT samples from the same patient were properly paired, a fingerprint analysis was performed running NGSCheckMate [43] on calls from HaplotypeCaller [44] (detailed below). Sequencing reads were aligned to the human reference genome GRCh37/hg19 using the Burrows–Wheeler Aligner (BWA v0.7.17) mapping tool [45], with setting ‘bwa mem -M’. Intermediate sam files were processed by Samtools v1.9 [46] and marked for duplicates by MarkDuplicates (Picard Toolkit v2.3.0, Broad Institute). Base quality recalibration was not performed. CollectAlignmentSummaryMetrics (Picard v2.3.0) was employed to estimate the sequencing depth and coverage for each sample, based on the alignments. Mosdepth v0.2.9 was used to calculate the mean read depth in target regions [47].

#### 4.6.2. Variant Calling and Filtering

Germline raw variants were called in tumor and NAT tissues by GATK4 HaplotypeCaller (v4.1.7), with default parameters and suggested inputs, including the Single Nucleotide Polymorphism Database (dbSNP, build 138) [48] as a resource for human variation. To obtain high-confidence catalogues of germline point mutations, we applied GATK VariantFiltration (v4.1.7) with the following options: quality by depth (QD < 2.0), Phred-scaled P-value using Fisher’s exact test for strand bias (FS > 60.0), mapping quality (MQ < 40.0), MappingQualityRankSumTest (MQRankSum < 12.5), ReadPosRankSumTest (ReadPosRankSum < 8.0), StrandOddsRatio (SOR > 4.0), and Phred scaled quality score (QUAL < 30.0).

In order to distinguish tumor-specific somatic mutations from germline variants, each tumor–organoid pair was compared to the matched NAT sample, used as the healthy control. However, NAT samples showed a mutation pattern very similar to those arising from tumor tissues, a condition that resulted in a low concordance among tumor–organoid pairs. Histologically normally NAT samples may contain their own somatic molecular alterations, infiltrating tumor cells, and/or cancerized cells. Considering these potential confounding sources of somatic variants when using breast NAT samples as control, we analyzed tumor and organoid samples within a control-free or tumor-only workflow. In the absence of eligible associated normal samples, we employed a virtual PoN of about 400 blood samples belonging to healthy and unrelated individuals, constructed at Broad Institute (available at gs://gatk-best-practices/somatic-b37/Mutect2-exome-panel.vcf), to help with the identification of recurrent sequencing artifactual and germline sites. Additionally, data about human population allele frequency (AF), from whole-exome or whole-genome sequencing of very large numbers of samples into the Genome Aggregation Database (gnomAD) [49], were used to exclude potential germline variants. A version of gnomAD stripped of all fields except VAF provided by Broad Institute was downloaded from gs://gatk-best-practices/somatic-b37/af-only-gnomad.raw.sites.vcf. Then, raw somatic mutations were called by providing tumor tissue and organoid sequencing data to MuTect2 GATK4 (v4.1.7) [50] in tumor-only mode, with setting employing the GATK best practices resources for the ‘--germline-resource’ and ‘--pon’ (panel of normals) arguments. The lists of high-confidence somatic mutations comprised the “PASS” flagged variants from FilterMutectCalls GATK4 (v4.1.7), with default parameter setting. Variants were annotated by Annovar algorithm [51], including information from cancer (i.e., COSMIC) [52] and population variant databases (i.e., dbSNP, 1000 Genomes project, Exome Aggregation Consortium) [53].

Concordance of mutation calls between tumor tissue and derived organoid was calculated using the Jaccard Index (J). For all mutations annotated by Annovar in the target region, the J index was calculated as follows:

J = C/(T + O − C)

where T is the number of events annotated in the tumor sample; O is the number of events annotated in the organoid; and C is the number of concordant events, shared in each tumor–organoid pairs. The percentage of J gives a measure of similarity between tumor and organoid samples from the same patient.

#### 4.6.3. Tumor Mutational Burden

The tumor mutational burden (TMB) in tumor tissue and organoid samples was defined as the total number of mutations with an allele frequency (VAF) ≥ 5% in gene coding regions, as well as the number of nonsynonymous mutations.

#### 4.6.4. Cancer-Associated Mutation Spectra Analysis

Mutational signatures were extracted by the non-negative matrix factorization (NMF) approach of the mutationalPatterns R package [54]. The MutationalPatterns fitting approach was then used to investigate the contribution of the thirty single-base substitution (SBS) signatures in the Catalogue Of Somatic Mutations In Cancer (COSMIC) compendium (v2) [55], in tumor tissue and organoid samples.

#### 4.6.5. CNV Detection

The somatic copy number variations in tumor tissue and organoid samples were identified using CNVkit v0.7.3 [56], from BWA aligned WES data, obtained as previously described. In the absence of suitable normal samples, we create a “flat” reference of neutral copy number (i.e., log2 0.0) for each probe from the target and antitarget interval files, within the CNVkit batch command with setting “–t” target.bed “–a” antitarget.bed “—access” access-5k-mappable.hg19.bed “--drop-low-coverage”. The bin-level GC-normalized log2 ratios (.cnr) were compared among tumor–organoid pair samples from the same individual.

All statistical analyses were performed within the R environment (v3.6.2).

#### 4.6.6. Data Availability

Sequencing data accession number will be made available during review. Data access requests will be evaluated by the bioethical committee of the Friuli Venezia Giulia region of Italy.

### 4.7. Drug Treatment of Organoids

Organoids were passaged as described above and allowed to grow for 5–7 days. Organoids were then harvested, and the smaller ones were isolated by recovering the supernatant after gentle centrifugation (16 g, 1 min). Then, 96-well Optiplates were coated with 20 μL of Matrigel and 20 μL of Matrigel/cells suspension were added on top (250 organoids/ well). After 2–3 days, three wells (technical replicates) were randomly assigned for each drug treatment: 10 µM 4-Hydroxytamoxifen (H7904, Sigma), 1 nM Docetaxel (S1148, Selleckchem), 500 nM Afatinib (S7810, Selleckchem), 100 nM Dasatinib (S1021, Selleckchem), 100 nM Atorvastatin (S5715, Selleckchem), ethanol, or dimethyl sulfoxide (DMSO). After 7 days of treatments, cell medium was removed and 100 μL of 1:1 CellTiter-Glo 3D Reagent (Promega)/culture media per well were added to measure ATP as a proxy for viable cells, following the manufacturer’s instructions. Luminescence reading was performed in an EnSpire^®^ multimode plate reader (Perkin Elmer). Data were analyzed using GraphPad Prism 6.

## 5. Conclusions

In conclusion, our study supports the usage of PDOs to model different types of breast cancer, to unveil intrinsic therapy-resistant subclones in heterogeneous carcinomas, and to explore new therapeutic strategies. We have demonstrated that these models can be used as in vitro platforms to assess cancer cells sensitivity to standard therapies and, in addition, to test combination treatments aimed at overcoming drug resistance. Our results support the usage of PDOs to capture different features of breast cancer, but they also highlight the need to further develop this methodology with the implementation of second-generation PDOs, improving media composition, and co-culturing tumor cells and non-neoplastic components of the tumor ecosystem (including immune cells) in engineered matrices mimicking the physical properties of the TME.

## Supplementary Materials

The following are available online at www.mdpi.com/xxx/s1, Figure S1: Correlation between BCs Ki-67 index and generation efficiency of derived organoids; Figure S2: Histological features of tumor cells in PDOs; Figure S3: Immunohistochemical analysis of NAT-derived organoids; Figure S4: Cytokeratin 19 expression in PDOs and in parental BCs; Figure S5: Cytokeratin 5/6 expression in PDOs and in parental BCs; Figure S6: Comparative analysis of WES data in BC tumor-organoid pairs; Figure S7: Effect of Afatinib on PDOs #30 viability; Figure S8: Negative control for YAP immunostaining; Table S1: Clinical information and organoids characteristics of selected BC cases; Table S2: Percentage of ER positive cells in parental breast cancers and derived models, divided by the molecular subtype; Table S3: Alignment metrics of WES data; Table S4: Total number of somatic mutations and concordance within tumor–organoid pairs; Table S5: List of somatic mutations identified by WES in each matched tumor and organoid model, across five cases, annotated by Annovar (Excel file); Table S6: List of antibodies, together with their specifications, used for immunohistochemistry and immunofluorescence analyses; Supplementary Methods: Extended protocol for the generation of organoids from breast cancer samples.

## References

[1] Dagogo-Jack I., Shaw A.T. (2018). Tumour heterogeneity and resistance to cancer therapies. Nat. Rev. Clin. Oncol..

[2] Zardavas D., Irrthum A., Swanton C., Piccart M. (2015). Clinical management of breast cancer heterogeneity. Nat. Rev. Clin. Oncol..

[3] Leight J.L., Drain A.P., Weaver V.M. (2017). Extracellular Matrix Remodeling and Stiffening Modulate Tumor Phenotype and Treatment Response. Annu. Rev. Cancer Biol..

[4] velaei K., Samadi N., Barazvan B., Soleimani Rad J. (2016). Tumor microenvironment-mediated chemoresistance in breast cancer. The Breast.

[5] Hirata E., Sahai E. (2017). Tumor Microenvironment and Differential Responses to Therapy. Cold Spring Harb. Perspect. Med..

[6] Bhimani J., Ball K., Stebbing J. (2020). Patient-derived xenograft models—the future of personalised cancer treatment. Br. J. Cancer.

[7] Marshall L.J., Triunfol M., Seidle T. (2020). Patient-Derived Xenograft vs. Organoids: A Preliminary Analysis of Cancer Research Output, Funding and Human Health Impact in 2014–2019. Animals.

[8] Drost J., Clevers H. (2018). Organoids in cancer research. Nat. Rev. Cancer.

[9] Huang L., Holtzinger A., Jagan I., BeGora M., Lohse I., Ngai N., Nostro C., Wang R., Muthuswamy L.B., Crawford H.C. (2015). Ductal pancreatic cancer modeling and drug screening using human pluripotent stem cell– and patient-derived tumor organoids. Nat. Med..

[10] Sachs N., de Ligt J., Kopper O., Gogola E., Bounova G., Weeber F., Balgobind A.V., Wind K., Gracanin A., Begthel H. (2018). A Living Biobank of Breast Cancer Organoids Captures Disease Heterogeneity. Cell.

[11] Yao Y., Xu X., Yang L., Zhu J., Wan J., Shen L., Xia F., Fu G., Deng Y., Pan M. (2020). Patient-Derived Organoids Predict Chemoradiation Responses of Locally Advanced Rectal Cancer. Cell Stem Cell.

[12] Goldhammer N., Kim J., Timmermans-Wielenga V., Petersen O.W. (2019). Characterization of organoid cultured human breast cancer. Breast Cancer Res..

[13] Kabos P., Haughian J.M., Wang X., Dye W.W., Finlayson C., Elias A., Horwitz K.B., Sartorius C.A. (2011). Cytokeratin 5 positive cells represent a steroid receptor negative and therapy resistant subpopulation in luminal breast cancers. Breast Cancer Res. Treat..

[14] Martincorena I., Roshan A., Gerstung M., Ellis P., Van Loo P., McLaren S., Wedge D.C., Fullam A., Alexandrov L.B., Tubio J.M. (2015). High burden and pervasive positive selection of somatic mutations in normal human skin. Science.

[15] Curtius K., Wright N.A., Graham T.A. (2018). An evolutionary perspective on field cancerization. Nat. Rev. Cancer.

[16] Trujillo K.A., Heaphy C.M., Mai M., Vargas K.M., Jones A.C., Vo P., Butler K.S., Joste N.E., Bisoffi M., Griffith J.K. (2011). Markers of fibrosis and epithelial to mesenchymal transition demonstrate field cancerization in histologically normal tissue adjacent to breast tumors. Int. J. Cancer.

[17] Casbas-Hernandez P., Sun X., Roman-Perez E., D’Arcy M., Sandhu R., Hishida A., McNaughton K.K., Yang X.R., Makowski L., Sherman M.E. (2015). Tumor Intrinsic Subtype Is Reflected in Cancer-Adjacent Tissue. Cancer Epidemiol. Biomark. Prev..

[18] Weinstein J.N., Collisson E.A., Mills G.B., Shaw K.R.M., Ozenberger B.A., Ellrott K., Shmulevich I., Sander C., Stuart J.M. (2013). The Cancer Genome Atlas Pan-Cancer analysis project. Nat. Genet..

[19] Chakravarty D., Gao J., Phillips S., Kundra R., Zhang H., Wang J., Rudolph J.E., Yaeger R., Soumerai T., Nissan M.H. (2017). OncoKB: A Precision Oncology Knowledge Base. JCO Precis. Oncol..

[20] Alexandrov L.B., Stratton M.R. (2014). Mutational signatures: The patterns of somatic mutations hidden in cancer genomes. Curr. Opin. Genet. Dev..

[21] Pleasance E., Titmuss E., Williamson L., Kwan H., Culibrk L., Zhao E.Y., Dixon K., Fan K., Bowlby R., Jones M.R. (2020). Pan-cancer analysis of advanced patient tumors reveals interactions between therapy and genomic landscapes. Nat. Cancer.

[22] Zanconato F., Cordenonsi M., Piccolo S. (2019). YAP and TAZ: A signalling hub of the tumour microenvironment. Nat. Rev. Cancer.

[23] Sorrentino G., Ruggeri N., Zannini A., Ingallina E., Bertolio R., Marotta C., Neri C., Cappuzzello E., Forcato M., Rosato A. (2017). Glucocorticoid receptor signalling activates YAP in breast cancer. Nat. Commun..

[24] Calvo F., Ege N., Grande-Garcia A., Hooper S., Jenkins R.P., Chaudhry S.I., Harrington K., Williamson P., Moeendarbary E., Charras G. (2013). Mechanotransduction and YAP-dependent matrix remodelling is required for the generation and maintenance of cancer-associated fibroblasts. Nat. Cell Biol..

[25] Sorrentino G., Ruggeri N., Specchia V., Cordenonsi M., Mano M., Dupont S., Manfrin A., Ingallina E., Sommaggio R., Piazza S. (2014). Metabolic control of YAP and TAZ by the mevalonate pathway. Nat. Cell Biol..

[26] Li X., Larsson P., Ljuslinder I., Öhlund D., Myte R., Löfgren-Burström A., Zingmark C., Ling A., Edin S., Palmqvist R. (2020). Ex Vivo Organoid Cultures Reveal the Importance of the Tumor Microenvironment for Maintenance of Colorectal Cancer Stem Cells. Cancers.

[27] Haughian J.M., Pinto M.P., Harrell J.C., Bliesner B.S., Joensuu K.M., Dye W.W., Sartorius C.A., Tan A.C., Heikkila P., Perou C.M. (2012). Maintenance of hormone responsiveness in luminal breast cancers by suppression of Notch. Proc. Natl. Acad. Sci. USA.

[28] Dongre A., Weinberg R.A. (2019). New insights into the mechanisms of epithelial–mesenchymal transition and implications for cancer. Nat. Rev. Mol. Cell Biol..

[29] Rosenbluth J.M., Schackmann R.C.J., Gray G.K., Selfors L.M., Li C.M.-C., Boedicker M., Kuiken H.J., Richardson A., Brock J., Garber J. (2020). Organoid cultures from normal and cancer-prone human breast tissues preserve complex epithelial lineages. Nat. Commun..

[30] Fiorini E., Veghini L., Corbo V. (2020). Modeling Cell Communication in Cancer With Organoids: Making the Complex Simple. Front. Cell Dev. Biol..

[31] Roerink S.F., Sasaki N., Lee-Six H., Young M.D., Alexandrov L.B., Behjati S., Mitchell T.J., Grossmann S., Lightfoot H., Egan D.A. (2018). Intra-tumour diversification in colorectal cancer at the single-cell level. Nature.

[32] Litchfield K., Stanislaw S., Spain L., Gallegos L.L., Rowan A., Schnidrig D., Rosenbaum H., Harle A., Au L., Hill S.M. (2020). Representative Sequencing: Unbiased Sampling of Solid Tumor Tissue. Cell Rep..

[33] Li L., Knutsdottir H., Hui K., Weiss M.J., He J., Philosophe B., Cameron A.M., Wolfgang C.L., Pawlik T.M., Ghiaur G. (2019). Human primary liver cancer organoids reveal intratumor and interpatient drug response heterogeneity. JCI Insight.

[34] Vlachogiannis G., Hedayat S., Vatsiou A., Jamin Y., Fernández-Mateos J., Khan K., Lampis A., Eason K., Huntingford I., Burke R. (2018). Patient-derived organoids model treatment response of metastatic gastrointestinal cancers. Science.

[35] Lambert A.W., Pattabiraman D.R., Weinberg R.A. (2017). Emerging Biological Principles of Metastasis. Cell.

[36] Pauli C., Hopkins B.D., Prandi D., Shaw R., Fedrizzi T., Sboner A., Sailer V., Augello M., Puca L., Rosati R. (2017). Personalized In Vitro and In Vivo Cancer Models to Guide Precision Medicine. Cancer Discov..

[37] Taccioli C., Sorrentino G., Zannini A., Caroli J., Beneventano D., Anderlucci L., Lolli M., Bicciato S., Del Sal G. (2015). MDP, a database linking drug response data to genomic information, identifies dasatinib and statins as a combinatorial strategy to inhibit YAP/TAZ in cancer cells. Oncotarget.

[38] Vennin C., Chin V.T., Warren S.C., Lucas M.C., Herrmann D., Magenau A., Melenec P., Walters S.N., del Monte-Nieto G., Conway J.R.W. (2017). Transient tissue priming via ROCK inhibition uncouples pancreatic cancer progression, sensitivity to chemotherapy, and metastasis. Sci. Transl. Med..

[39] Hirata E., Girotti M.R., Viros A., Hooper S., Spencer-Dene B., Matsuda M., Larkin J., Marais R., Sahai E. (2015). Intravital Imaging Reveals How BRAF Inhibition Generates Drug-Tolerant Microenvironments with High Integrin β1/FAK Signaling. Cancer Cell.

[40] Allison K.H., Hammond M.E.H., Dowsett M., McKernin S.E., Carey L.A., Fitzgibbons P.L., Hayes D.F., Lakhani S.R., Chavez-MacGregor M., Perlmutter J. (2020). Estrogen and Progesterone Receptor Testing in Breast Cancer: American Society of Clinical Oncology/College of American Pathologists Guideline Update. Arch. Pathol. Lab. Med..

[41] Wolff A.C., Hammond M.E.H., Allison K.H., Harvey B.E., Mangu P.B., Bartlett J.M.S., Bilous M., Ellis I.O., Fitzgibbons P., Hanna W. (2018). Human Epidermal Growth Factor Receptor 2 Testing in Breast Cancer: American Society of Clinical Oncology/College of American Pathologists Clinical Practice Guideline Focused Update. Arch. Pathol. Lab. Med..

[42] Dowsett M., Nielsen T.O., A’Hern R., Bartlett J., Coombes R.C., Cuzick J., Ellis M., Henry N.L., Hugh J.C., Lively T. (2011). Assessment of Ki67 in Breast Cancer: Recommendations from the International Ki67 in Breast Cancer Working Group. JNCI J. Natl. Cancer Inst..

[43] Lee S., Lee S., Ouellette S., Park W.-Y., Lee E.A., Park P.J. (2017). NGSCheckMate: Software for validating sample identity in next-generation sequencing studies within and across data types. Nucleic Acids Res..

[44] Poplin R., Ruano-Rubio V., DePristo M.A., Fennell T.J., Carneiro M.O., Van der Auwera G.A., Kling D.E., Gauthier L.D., Levy-Moonshine A., Roazen D. (2017). Scaling accurate genetic variant discovery to tens of thousands of samples. bioRxiv.

[45] Li H., Durbin R. (2009). Fast and accurate short read alignment with Burrows-Wheeler transform. Bioinformatics.

[46] Li H., Handsaker B., Wysoker A., Fennell T., Ruan J., Homer N., Marth G., Abecasis G., Durbin R. (2009). The Sequence Alignment/Map format and SAMtools. Bioinformatics.

[47] Pedersen B.S., Quinlan A.R. (2018). Mosdepth: Quick coverage calculation for genomes and exomes. Bioinformatics.

[48] Sherry S.T. (2001). dbSNP: The NCBI database of genetic variation. Nucleic Acids Res..

[49] Karczewski K.J., Francioli L.C., Tiao G., Cummings B.B., Alföldi J., Wang Q., Collins R.L., Laricchia K.M., Ganna A., Birnbaum D.P. (2020). The mutational constraint spectrum quantified from variation in 141,456 humans. Nature.

[50] Benjamin D., Sato T., Cibulskis K., Getz G., Stewart C., Lichtenstein L. (2019). Calling Somatic SNVs and Indels with Mutect2. bioRxiv.

[51] Wang K., Li M., Hakonarson H. (2010). ANNOVAR: Functional annotation of genetic variants from high-throughput sequencing data. Nucleic Acids Res..

[52] Alexandrov L.B., Jones P.H., Wedge D.C., Sale J.E., Campbell P.J., Nik-Zainal S., Stratton M.R. (2015). Clock-like mutational processes in human somatic cells. Nat. Genet..

[53] Auton A., Abecasis G.R., Altshuler D.M., Durbin R.M., Bentley D.R., Chakravarti A., Clark A.G., Donnelly P., Eichler E.E., Flicek P. (2015). A global reference for human genetic variation. Nature.

[54] Blokzijl F., Janssen R., van Boxtel R., Cuppen E. (2018). MutationalPatterns: Comprehensive genome-wide analysis of mutational processes. Genome Med..

[55] Alexandrov L.B., Nik-Zainal S., Wedge D.C., Aparicio S.A.J.R., Behjati S., Biankin A.V., Bignell G.R., Bolli N., Borg A., Børresen-Dale A.-L. (2013). Signatures of mutational processes in human cancer. Nature.

[56] Talevich E., Shain A.H., Botton T., Bastian B.C. (2016). CNVkit: Genome-Wide Copy Number Detection and Visualization from Targeted DNA Sequencing. PLOS Comput. Biol..

