# Supplementary material for: Breast Cancer Organoids Model Patient-Specific Response to Drug Treatment
Source: Cancers (Basel). 2020 Dec 21;12(12):3869. doi: 10.3390/cancers12123869 (PMC7770601; doi:10.3390/cancers12123869)
Supplement: Supplementary file 1 [file cancers-12-03869-s001.zip › cancers-983905_Supplementary_Proofreading_EC.docx]

Supplementary Materials


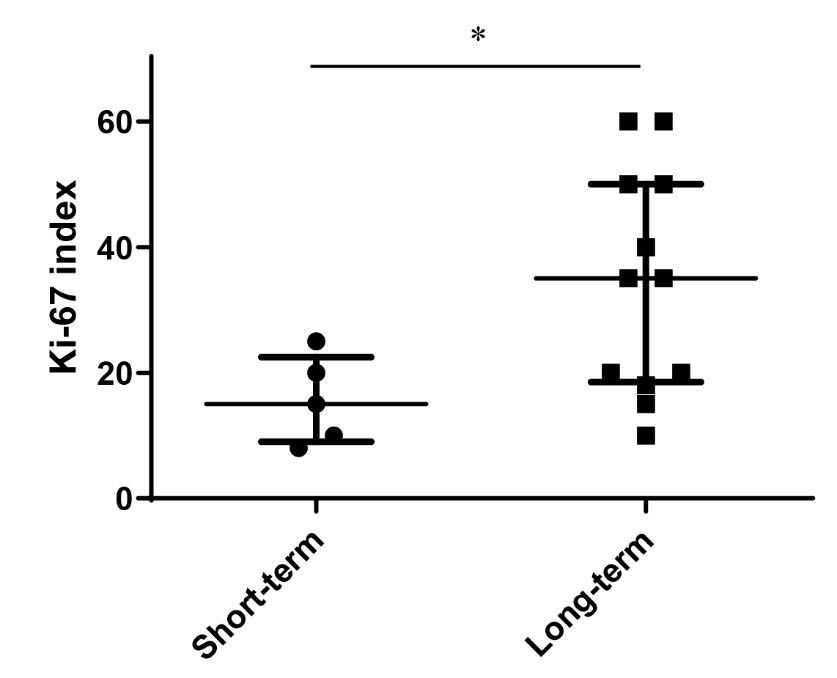


**Figure S1.** Correlation between BCs Ki-67 index and generation efficiency of derived organoids. Dot plot representing the Ki-67 index (% of positive cells) of primary BC compared to the proliferation of derived organoids. Ki-67 index is calculated as percentage of tumor cells that positively stained for Ki-67 in FFPE tissues, using an anti-Ki-67 antibody. Long-term organoids are defined as PDOs maintained for more than 4 weeks in culture (passages ≥4), a threshold empirically determined based on the possibility to perform PDOs histological characterization and cryopreservation. Shown are also median values and interquantile ranges for each group; * *p* <0.05, two-tailed Mann-Whitney nonparametric test.


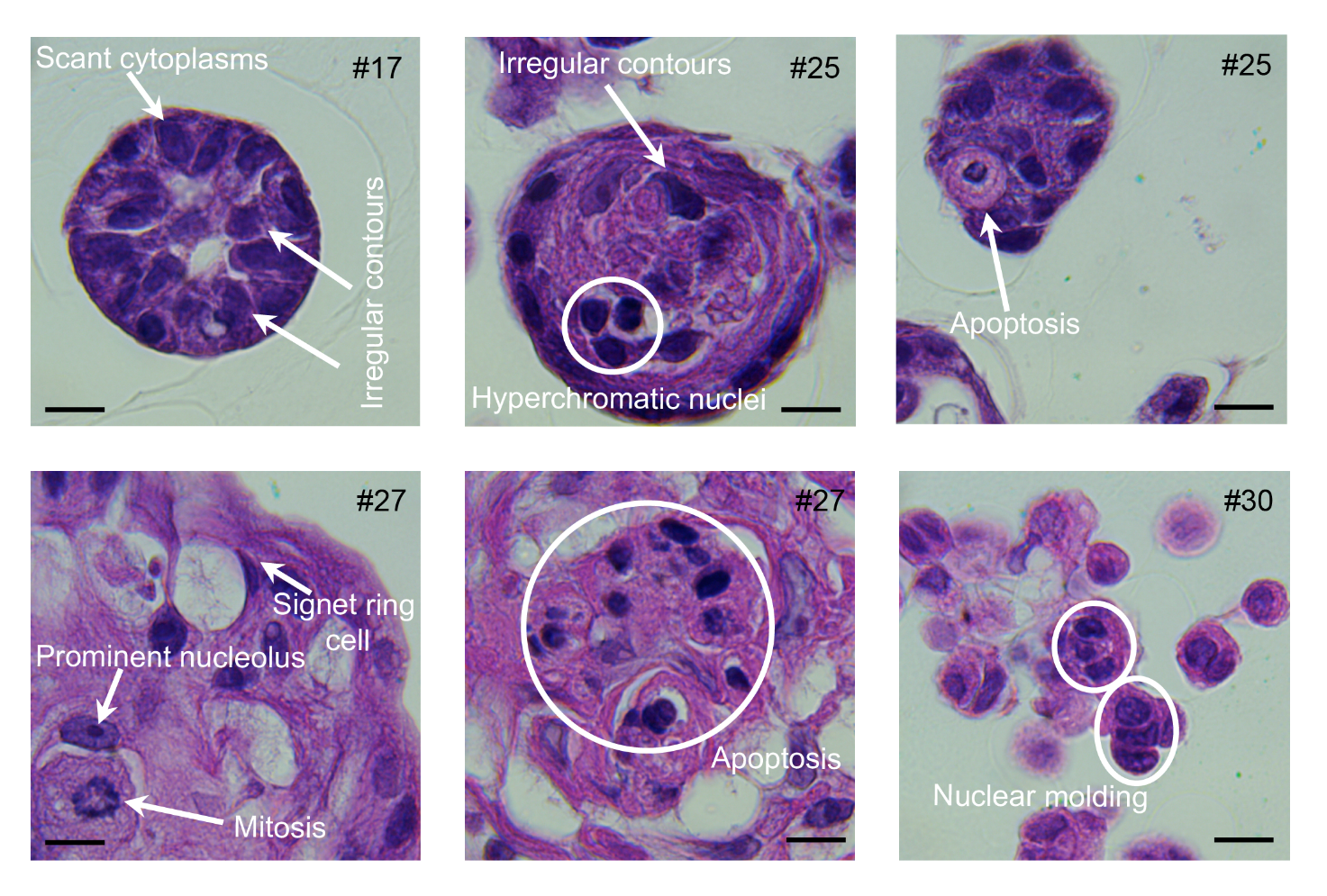


**Figure S2.** Histological features of tumor cells in PDOs. High power magnifications of BC organoids showing characteristic histological features of tumor cells. From left to right: cells with scant cytoplasms, nuclei with irregular contours, hyperchromatic nuclei, apoptotic cells, mitotic cell, nucleus with prominent nucleolus, vacuole formation (signet ring cell), and nuclear molding. Scale bar, 20 μm.


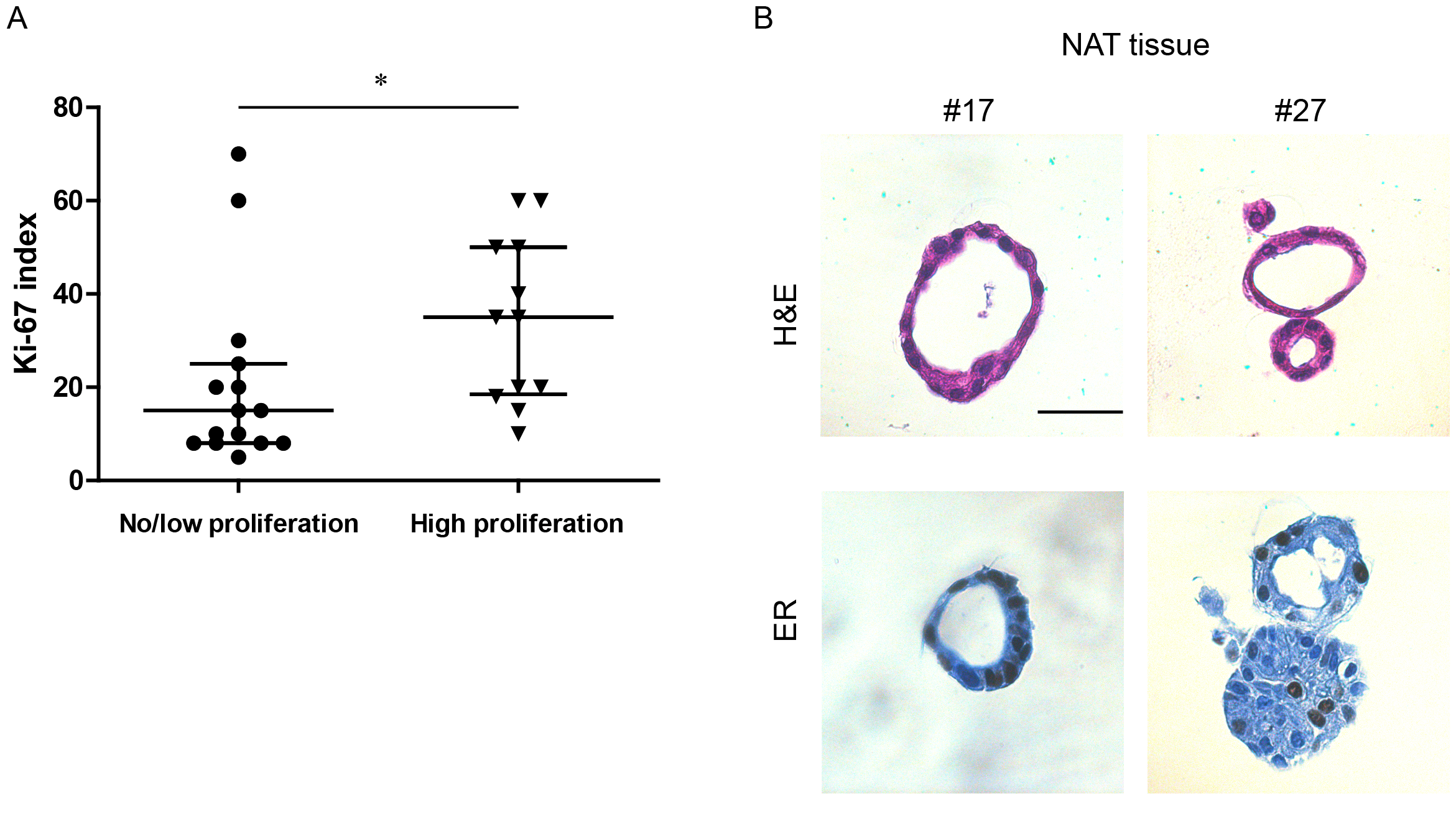


**Figure S3.** Immunohistochemical analysis of NAT-derived organoids. Representative images of hematoxylin/eosin staining (H&E) and immunohistochemical analysis of ER on sections of organoids derived from NAT tissue. Scale bar, 100 μm.


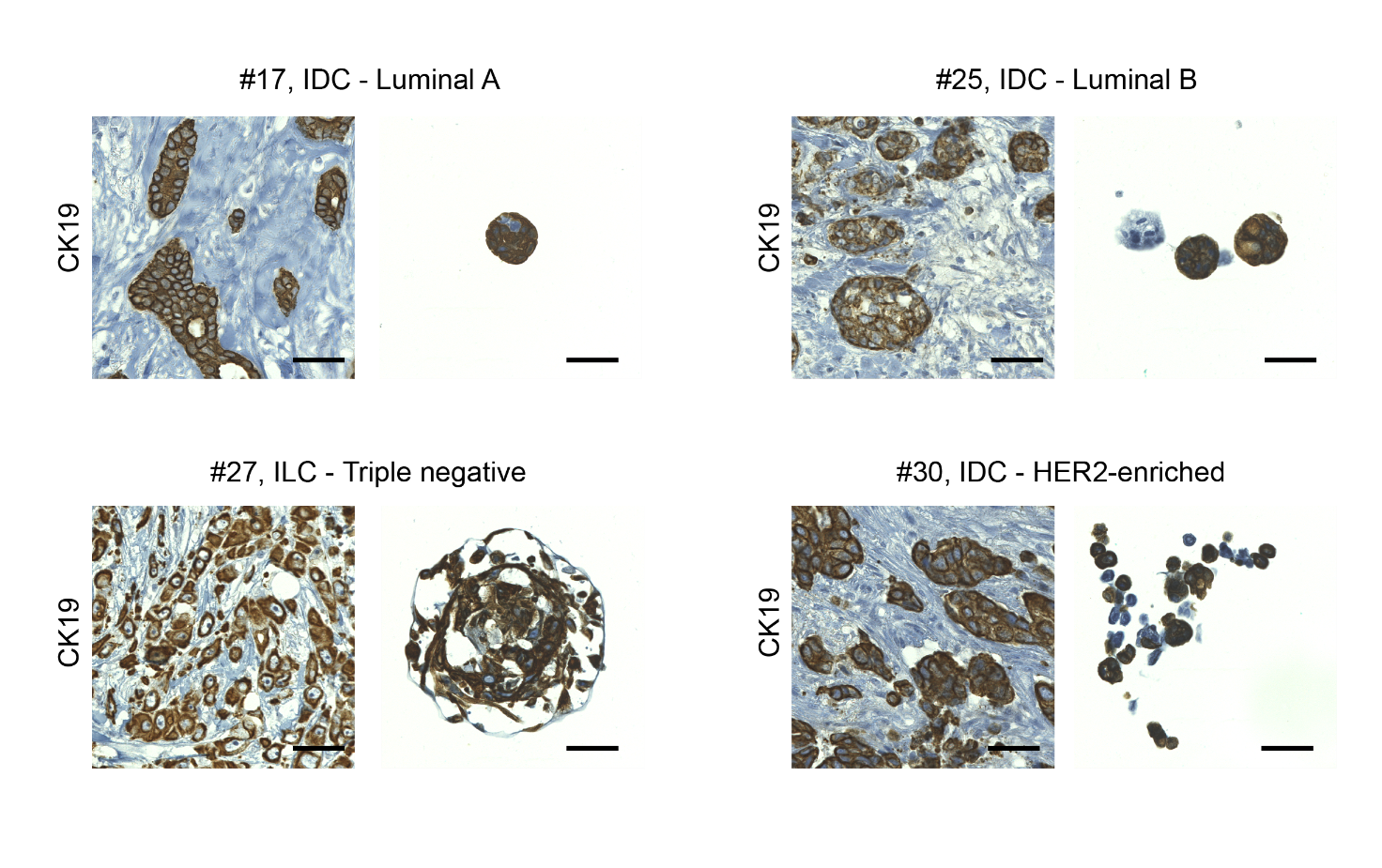


**Figure S4.** Cytokeratin 19 expression in PDOs and in parental BCs. Representative images of immunohistochemical analyses on sections of BCs and derived organoids. From left to right and from the top to the bottom are shown examples of luminal A, luminal B, triple negative, and HER2-enriched BCs respectively; CK19, cytokeratin 19; Scale bar, 100 μm.


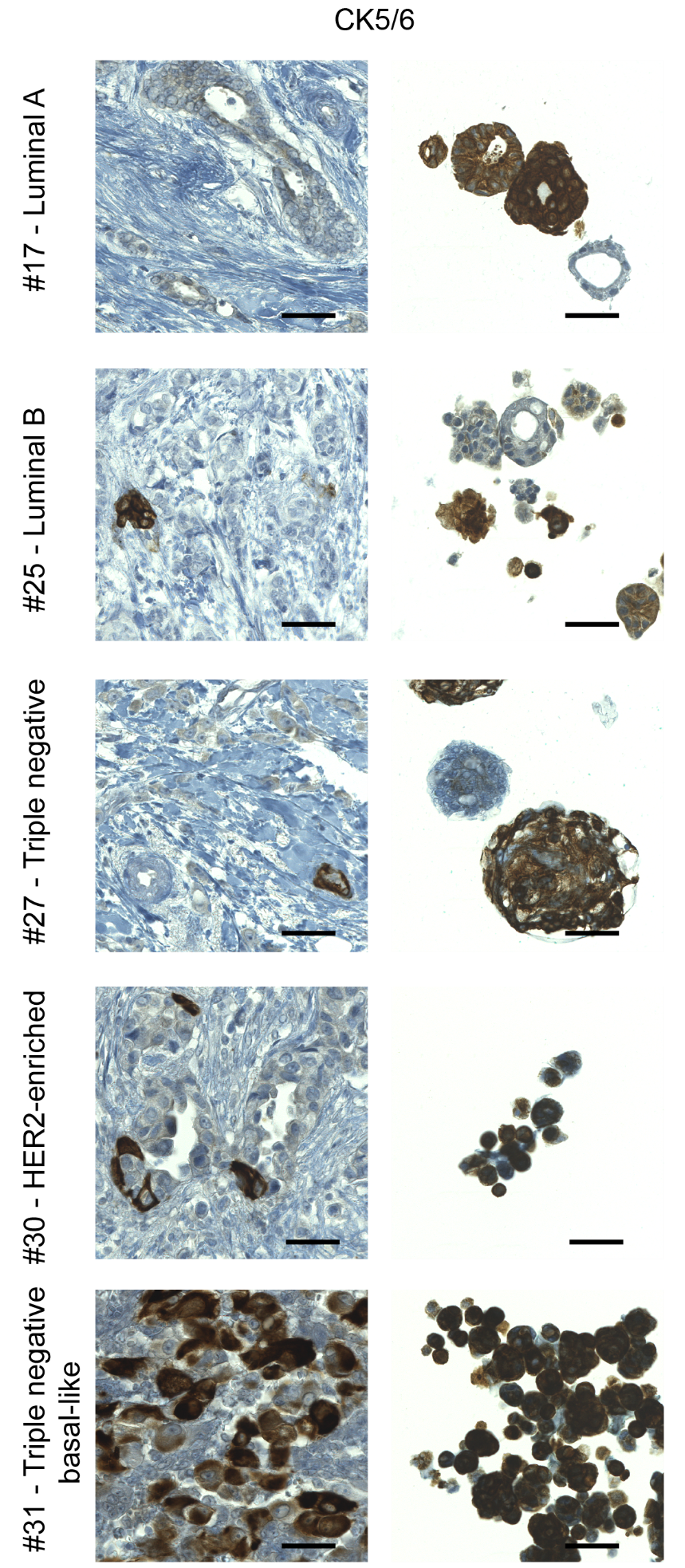


**Figure S5.** Cytokeratin 5/6 expression in PDOs and in parental BCs. Representative images of immunohistochemical analyses of cytokeratin 5/6 on sections of BCs (left) and derived organoids (right). From the top to the bottom are shown examples of luminal A, luminal B, triple negative, HER2-enriched and triple negative basal-like BCs; Ck5/6, cytokeratin 5/6; Scale bar, 100 μm.


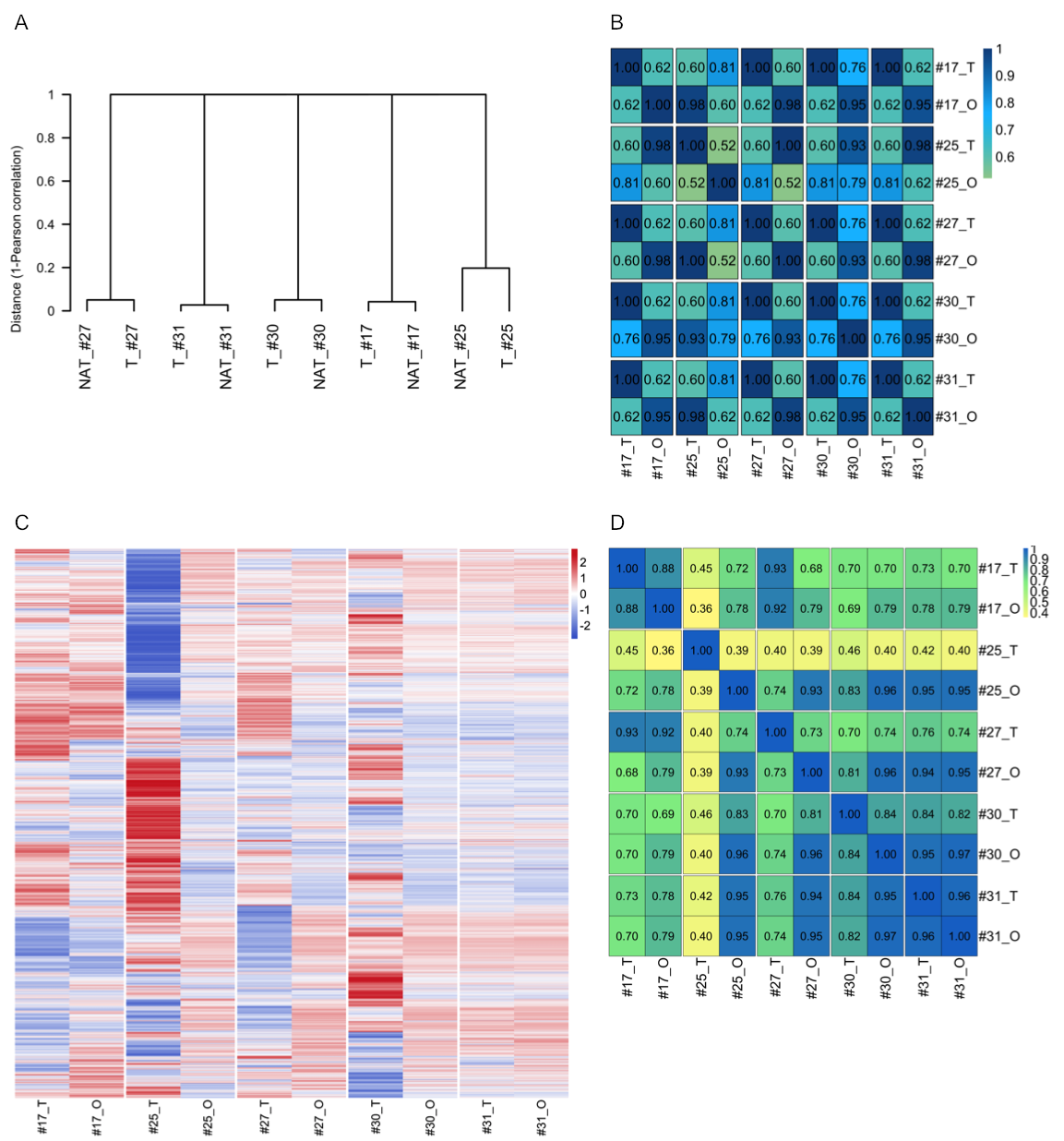


**Figure S6.** Comparative analysis of WES data in BC tumor-organoid pairs. (**a**) Fingerprint analysis of WES samples. The dendrogram output for five primary breast cancer and normal-appearing adjacent-to-tumor (NAT) samples, based on 1 - VAF (Pearson) correlation as the distance measure. All tumor-NAT pairs show a proper sample matching. The numbers in the sample labels represent the case IDs. (**b**) Pairwise Spearman’s correlation referring to the mutation spectra among tumor and organoid samples is shown. (**c**) Heat map comparing copy number variations across tumor-organoid pairs in a log2 scale. Red colors indicate gains, and blue colors indicate losses. (**d**) Pairwise Spearman’s correlation referring to copy number variations among tumor and organoid samples is shown.


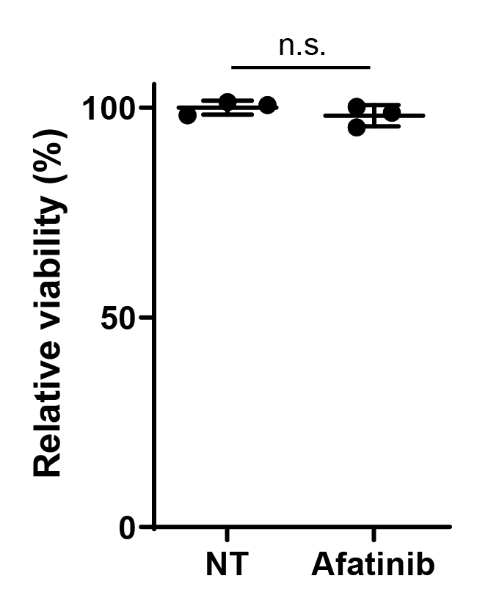


**Figure S7.** Effect of Afatinib on PDOs #30 viability. Dot plot showing relative viability of BC organoids treated with 0.5 μM Afatinib or with solvent (NT, DMSO) for 4 days. Data shown are the means ± s.d. of n=3 independent experiments, n.s. not significant; t-test.


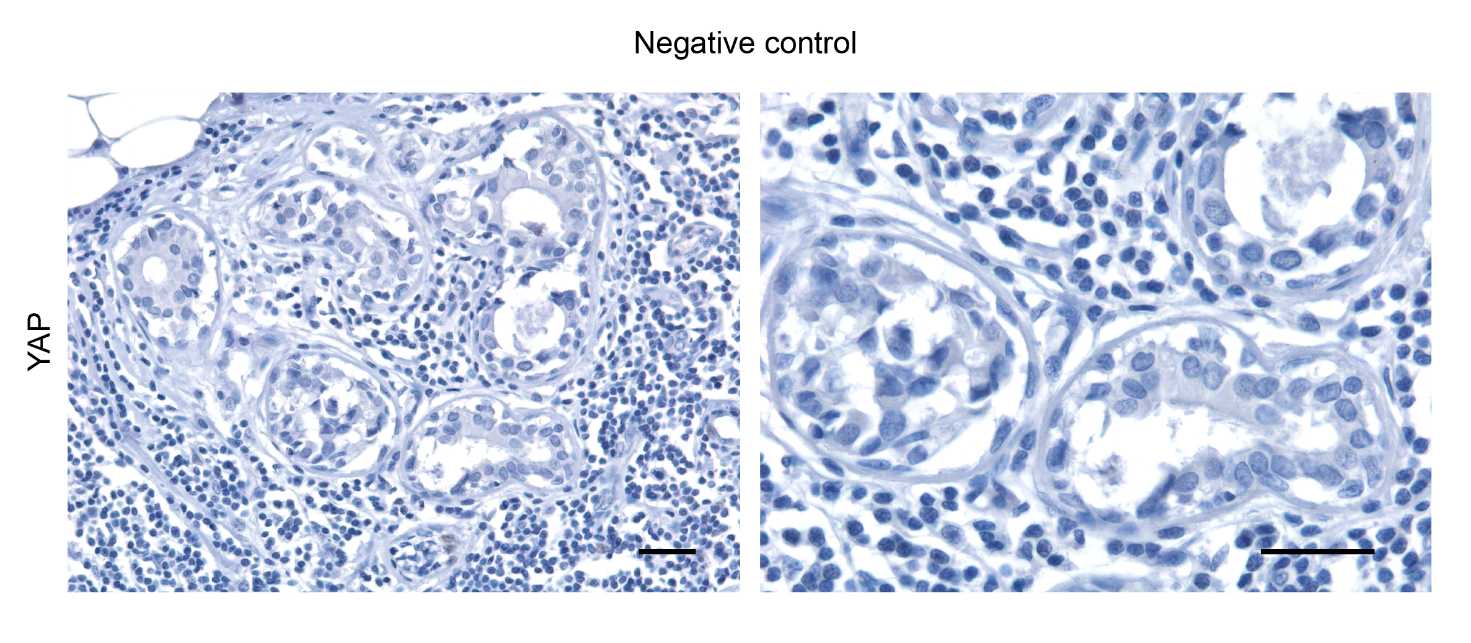


**Figure S8.** Negative control for YAP immunostaining. As a negative control for YAP immunostaining, following antigenic unmasking, breast cancer tissue sections (that resulted positively immunoreactive for YAP) were incubated with the secondary antibody and peroxidase chromogenic substrate, without the addition of the anti-YAP specific antibody. Under these conditions, no signal (either cytoplasmatic or nuclear) is observed in the control sections. Scale bar, 100 μm.

**Table S1.** Clinical information and organoids characteristics of selected BC cases.

| **Patient** | | **Tumor** | | | | **Organoids from cancer tissue** | | **Organoids from NAT tissue** |
| --- | --- | --- | --- | --- | --- | --- | --- | --- |
| **#** | **Age**  **(years)** | **Histological subtype** | **Molecular subtype** | **Grade** | **Ki-67 index** | **Organoids establishment and n. of passages (p)** | **Histological match** | **Organoids establishment and n. of passages (p)** |
| 1 | 43 | IDC | Lum A | G2 | 10 | - |  | p1 |
| 2 | 89 | IDC | Lum B | G3 | 30 | - |  | - |
| 3 | 73 | IDC | Lum A | G2 | 18 | - |  | no biopsy |
| 4 | 50 | IDC | TNBC | G3 | 40 | - |  | no biopsy |
| 5 | 74 | ILC | Lum A |  | 8 | - |  | - |
| 6 | 52 | ILC | Lum A |  | 10 | - |  | - |
| 7 | 50 | IDC | Lum A | G1 | 8 | - |  | - |
| 8 | 66 | IDC | Lum A | G3 | 60 | - |  | - |
| 9 | 70 | IDC | Lum A | G2 | 40 | p4 | NA | - |
|  |  |  |  |  |  | 1/9 | NA:1 | 1/7 |
| *10 | 65 | IDC | Lum A | G1 | 8 | - |  | - |
| 11 | 79 | IDC | Lum A | G1 | 8 | - |  | p1 |
| 12 | 59 | IDC | Lum A | G2 | 15 | p3 | No^†^ | p2 |
| 13 | 46 | IDC | Lum A | G2 | 20 | p3 | NA | p1 |
| 14 | 75 | IDC | Lum B | G3 | 25 | p3 | Yes | p2 |
| 15 | 88 | IDC | Lum A | G2 | 8 | p3 | Yes | p1 |
| 16 | 66 | IDC | Lum A | G2 | 20 | - |  | - |
| 17 | 60 | IDC | Lum A | G1 | 15 | p6 | Yes | p1 |
| 18 | 48 | IDC | TNBC | G3 | 60 | p5 | Yes | p1 |
| 19 | 53 | IDC | Lum B | G3 | 50 | p5 | Yes | p1 |
| 20 | 73 | ILC | Lum A |  | 18 | p5 | NA | p1 |
| 21 | 60 | ILC | Lum A |  | 10 | p4 | No^†^ | p2 |
| 22 | 71 | ILC | TNBC |  | 5 | - |  | p1 |
| 23 | 59 | IDC | Lum A | G1 | 10 | p3 | NA | p2 |
| 24 | 83 | IDC | HER2-enr | G3 | 30 | - |  | p1 |
| 25 | 40 | IDC | Lum B | G3 | 35 | p7 | Yes | p1 |
| 26 | 56 | IDC | Lum A | G2 | 20 | p6 | Yes | p2 |
| 27 | 87 | ILC | TNBC |  | 20 | p6 | Yes | p1 |
| 28 | 77 | IDC | Lum A | G2 | 15 | - |  | - |
| 29 | 98 | IDC | TNBC | G3 | 70 | - |  | no biopsy |
| 30 | 63 | IDC | HER2-enr | G3 | 60 | p7 | Yes (Her2-) | p1 |
| 31 | 87 | ILC | TNBC | G3 | 35 | p8 | Yes | p2 |
| 32 | 65 | IDC | Lum B | G3 | 50 | p7 | No^†^ | - |
|  |  |  |  |  |  | 16/23 | Yes:10; No:3; NA:3 | 18/22 |

Table lists clinical information and organoids characteristics of selected BC cases. For histological subtype determination (ductal vs. lobular carcinoma), expression and localization of E-cadherin and p120 catenin were analyzed in breast cancer tissues. -, no organoid generation; NA (not assessed), due to technical problems during sample preparation. *, starting from this case, plate coating with Matrigel before seeding has been included in the methodology. ^†^, ER expression loss and no concordance in cytokeratins expression.

**Table S2.** Percentage of ER positive cells in parental breast cancers and derived models, divided by the molecular subtype.

|  | **Patient #** | **Tumor (%)** | **Organoids (%)** |
| --- | --- | --- | --- |
| Luminal A | 9 | 98 | NA |
|  | 12 | 95 | 5 |
|  | 13 | 95 | NA |
|  | 15 | 95 | 40 |
|  | 17 | 95 | 70 |
|  | 20 | 95 | NA |
|  | 21 | 90 | 10 |
|  | 23 | 98 | NA |
|  | 26 | 98 | 60 |
| Luminal B | 14 | 95 | 50 |
|  | 19 | 80 | 40 |
|  | 25 | 90 | 30 |
|  | 32 | 95 | 5 |
| HER2-enriched | 30 | 90 | 5 |
| Triple negative | 18 | 0 | 0 |
|  | 27 | 0 | 0 |
|  | 31 | 0 | 0 |

NA: not assessed.

**Table S3.** Alignment metrics of WES data.

| **#** | **Sample Type** | **TOTAL_READS** | **PF_READS** | **PF_READS_ALIGNED** | **PF_ALIGNED_BASES** | **PF_HQ_ALIGNED_READS** | **PF_HQ_ALIGNED_BASES** | **MEAN_READ_LENGTH** | **Coverage (%)** | **Mean Depth** |
| --- | --- | --- | --- | --- | --- | --- | --- | --- | --- | --- |
| 17 - Lum A | **NAT** | 152215488 | 152215488 | 152190901 | 22735547410 | 148440162 | 22194079538 | 150 | 99 | 115.42 |
|  | **Organoid** | 192827369 | 192827369 | 192758807 | 28685077129 | 187274530 | 27910409169 | 149 | 98 | 137.08 |
|  | **Tumor** | 120191042 | 120191042 | 120158657 | 17858916459 | 117082278 | 17417144986 | 149 | 96 | 92.97 |
| 25 - Lum B | **NAT** | 26779386 | 26779386 | 26666022 | 3941139330 | 25362827 | 3755844208 | 149 | 66 | 15.03 |
|  | **Organoid** | 127670390 | 127670390 | 127647235 | 19096108353 | 124346491 | 18621570773 | 150 | 97 | 100.14 |
|  | **Tumor** | 32461332 | 32461332 | 32428835 | 4787025857 | 31048619 | 4588547903 | 149 | 74 | 18.92 |
| 27 - TN | **NAT** | 136952510 | 136952510 | 136926258 | 20351731836 | 133110238 | 19820146701 | 149 | 98 | 83.86 |
|  | **Organoid** | 176233079 | 176233079 | 176119103 | 26198694303 | 171144860 | 25516361864 | 149 | 99 | 134.13 |
|  | **Tumor** | 121554255 | 121554255 | 121526184 | 18122434745 | 118060765 | 17622484565 | 150 | 96 | 91.58 |
| 30 - HER2 | **NAT** | 143415702 | 143415702 | 143392430 | 21334031227 | 139406135 | 20763315687 | 149 | 98 | 107.41 |
|  | **Organoid** | 152601420 | 152601420 | 152476112 | 22802025625 | 148377955 | 22212642874 | 150 | 98 | 121.88 |
|  | **Tumor** | 116724626 | 116724626 | 116708575 | 17363623993 | 113605685 | 16918232203 | 149 | 97 | 85.15 |
| 31 - TN | **NAT** | 180475771 | 180475771 | 180440977 | 26870801331 | 175705487 | 26189927687 | 149 | 99 | 139.32 |
|  | **Organoid** | 130814244 | 130814244 | 130623248 | 19527418795 | 127107612 | 19025323429 | 150 | 98 | 104.05 |
|  | **Tumor** | 130526028 | 130526028 | 130507527 | 19423196900 | 127037929 | 18924840749 | 149 | 98 | 100.2 |

Table lists the alignment metrics for both first and second reads in a pair, produced by CollectAlignmentSummaryMetrics package, for each sample. The percentage of target covered with at least 20× (Coverage (%)) and the mean read depth (Mean Depth), estimated by Mosdepth, are also reported for each sample. NAT: normal-appearing adjacent-to-tumor. PF: Passing Illumina's filter. TN: triple negative.

**Table S4.** Total number of somatic mutations and concordance within tumor-organoid pairs.

| **#** | **Type** | **# Mutations** | **Concordance (%)** | **% Tumor mutations in organoid** | **# Mutations in protein coding regions (mut/Mb)** | **# Nonsynonymous mutations (mut/Mb)** |
| --- | --- | --- | --- | --- | --- | --- |
| 17 - Lum A | Concordant | 1,111 |  |  | - | - |
|  | Organoid only | 515 | 43 | 54.3 | 256 (4.3) | 176 (2.9) |
|  | Tumor only | 934 |  |  | 337 (5.6) | 235 (3.9) |
| 25 - Lum B | Concordant | 370 |  |  | - | - |
|  | Organoid only | 1,130 | 2 | 4.1 | 231 (3.9) | 157 (2.6) |
|  | Tumor only | 8,549 |  |  | 2,587 (43.1) | 2,178 (36.3) |
| 27 - TN | Concordant | 1,035 |  |  | - | - |
|  | Organoid only | 748 | 37 | 47.7 | 189 (3.2) | 123 (2.1) |
|  | Tumor only | 1,133 |  |  | 328 (5.5) | 239 (4.0) |
| 30 - HER2 | Concordant | 926 |  |  | - | - |
|  | Organoid only | 471 | 13 | 11.3 | 185 (3.1) | 123 (2.1) |
|  | Tumor only | 7,253 |  |  | 1,227 (20.5) | 853 (14.2) |
| 31 - TN | Concordant | 1,216 |  |  | - | - |
|  | Organoid only | 486 | 38 | 41.6 | 294 (4.9) | 207 (3.5) |
|  | Tumor only | 1,710 |  |  | 393 (6.6) | 269 (4.5) |

Total number of somatic mutations (# Mutations) specifically identified in tumor and organoid samples, as well as the number of mutations shared within tumor-organoid pairs (Concordant) are listed. The percentage of tumor-organoid shared mutations in target regions, and tumor-specific mutations recapitulating in matched organoid, are reported for each case. Tumor mutational burden (TMB), as the total number of mutations in protein coding regions or the number of nonsynonymous mutations, is reported for each tumor and organoid sample. mut/Mb: mutation number / megabase. TN: triple negative.

**Table S5.** List of somatic mutations identified by WES in each matched tumor and organoid model, across five cases, annotated by Annovar. Note: separate Excel file.

**Table S6.** List of antibodies, together with their specifications, used for immunohistochemistry and immunofluorescence analyses.

| **Target** | **Provider** | **Catalog number** | **Clone name** | **Dilution** |
| --- | --- | --- | --- | --- |
| Estrogen receptor (ER) | Ventana, Roche Diagnostics | 790-4324 | SP1 | Pre-diluted |
| Progesterone receptor (PR) | Ventana, Roche Diagnostics | 790-2223 | 1E2 | Pre-diluted |
| HER2 | Ventana, Roche Diagnostics | 790-2991 | 4B5 | Pre-diluted |
| Ki-67 | Ventana, Roche Diagnostics | 790-4286 | 30-9 | Pre-diluted |
| Cytokeratin 19 (CK19) | Ventana, Roche Diagnostics | 760-4281 | A53-B/A2.26 | Pre-diluted |
| Cytokeratin 5/6 (CK5/6) | Ventana, Roche Diagnostics | 790-4554 | D5/16B4 | Pre-diluted |
| E-cadherin | Ventana, Roche Diagnostics | 760-4440 | EP700Y | Pre-diluted |
| p120-catenin | Ventana, Roche Diagnostics | 790-4517 | 98 | Pre-diluted |
| YAP (IHC) | Santa Cruz | SC-101199 | 63.7 | 1:50 |
| YAP (IF) | Abcam | ab52771 | EP1674Y | 1:200 |
| Vinculin | Sigma | v4505 | V284 | 1:500 |

Supplementary Methods

Extended protocol for the generation of organoids from breast cancer samples.

1. **Cell culture media, reagents and solutions**

- Human breast samples (cancer and normal tissue)
- Penicillin-Streptomycin Solution 100X (Euroclone, cat. no. ECB3001D)
- Primocin (Invivogen, cat.no. Ant-pm-1)
- Collagenase A (Roche, cat. no. 11088793001)
- FGF7 (Peprotech, cat. no. 100-19)
- FGF10 (Peprotech, cat.,no 100-26)
- Noggin (Peprotech, cat. no. 120-10C)
- Heregulin β-1 (Peprotech, cat. no. 100-03)
- R-spondin 3 (R&D system, cat. no. 3500-RS-025/CF)
- EGF (Cell Guidance Systems, cat. no. GFH26)
- A83-01 (Tocris, cat. no. 2939)
- Y-27632 (Selleckchem, cat. no. S1049)
- SB202190 (Sigma-Aldrich, cat. no.S7067)
- B27 supplement (Gibco, cat. no. 17504-44)
- N-Acetylcysteine (Sigma-Aldrich, cat. no. A9165)
- Nicotinamide (Sigma-Aldrich, cat. no. N0636)
- GlutaMax100X (Invitrogen, cat. no. 12634-034)
- Hepes (Invitrogen, cat. no. 15630-056)
- PBS-1× (Life Technologies, cat. no. 14190-094)
- DMSO (Sigma-Aldrich, cat. no. D8418)
- FBS (Euroclone, cat. no. ECS0180L)
- Advanced DMEM/F-12 (Ad-DF, Gibco cat. no. 12634-010)
- Matrigel Growth Factor Reduced (BD, cat. no. 354230)
- HBSS Hank’s Balanced Salt Solution modified, without Ca, Mg and Phenol Red (Lonza, cat. no. 04-315Q)
- Ammonium chloride Solution (Stemcell, cat. no. 07800)
- Cell recovery solution (Corning, cat. no. 354253)
- Dispase (Stemcell, cat. no. 07913)
- TrypLE Express 1X (Gibco, cat. no. 12605-010)

1. **Equipment**

- Cell culture incubator with 5% CO_2_, 37°C
- CKX31 Inverted Microscope, Olympus Life Sciences
- High-speed centrifuge (Eppendorf centrifuge 5810R)
- Pipette aid, serological pipets (Euroclone cat no. EPS05N; EPS10N)
- Pipettes
- Pipette tips
- Conical centrifuge tubes (Euroclone, cat. no. ET5015B; ET5050B)
- Microcentrifuge tubes (Euroclone, cat. no. ET3415)
- 100 mm Petri dish (Greiner Bio-one, cat. no. 633181)
- Carbon steel scalpel blades (Albion, cat. no. 03393)
- 100-µm Cell strainer (Falcon, cat. no. 352360)
- 24-Well adherent culture plate (Euroclone, cat. no. ET3024)
- 2-mL Cryogenic vials (VWR, cat. no. 479-0287)
- Corning CoolCell LX Cell Freezing Container (Sigma-Aldrich, cat. no. CLS432002-1EA)
- Ice

1. **Reagent setup**

- **Transport medium** Ad-DF medium supplemented with 5% FBS, 1X Penicillin/Streptomycin, 10mM Hepes.
- **Digestion solution** 1.6 U/mL collagenase A in transport medium. Sterile filtered. This solution should be freshly prepared and used immediately. For each sample at least 5mL of this solution should be prepared.
- **Ad-DF basal medium** Ad-DF supplemented with 1X Glutamax, 50 µg/mL Primocin, 1X Penicillin/Streptomycin, 10mM Hepes.
- **Human breast organoid medium** Human breast organoid medium (for normal and cancer specimen) is Ad-DF basal medium supplemented with:

| **Medium component** | **Final concentration** |
| --- | --- |
| B27 supplement | 1X |
| Nicotinamide | 5 mM |
| N-Acetylcystein | 1.25 mM |
| R-spondin 3 | 250 ng/mL |
| Heregulin β-1 | 5 nM |
| Noggin | 100 ng/mL |
| FGF-7 | 5 ng/mL |
| FGF-10 | 20 ng/mL |
| EGF | 5 ng/mL |
| A83-01 | 500 nM |
| SB202190 | 500 nM |
| Y-27632* | 5 μM |

* if single cells are plated, add it for the first three days of culture

- **Cell freezing solution** This solution is prepared by adding 10% of DMSO in FBS.

1. **Procedure**
   1. **Collection of fresh biopsies**

Fresh material from surgically resected breast primary cancers and the corresponding healthy tissue are collected from the operating theatre of the hospital. Punch biopsies of fresh breast tumor and normal breast tissue are collected in cold Transport medium and are delivered on ice to the research laboratory. At least 1 cm^3^ of tissue should be collected and processed within 6 hours.

- 1. **Breast tissues dissociation**

**!CRITICAL** For the following steps precoat all the tubes, the pipettes and tips with FBS to avoid attachment of the tissue to the plastic.

- - 1. Transfer the tube containing the breast tissue to the biological safety cabinet. Aspirate the medium and place the tissue in a sterile 100-mm Petri dish (untreated dish to avoid cell attachment) ***(Figure 1)***. Mince it into 1-3 mm^3^ pieces using scalpels ***(Figure 2)****.*
    2. At this step it is possible to proceed with tissue digestion or minced tissue can be store at -80°C (up to 3 months).

For tissue digestion, transfer the material in a 50mL tube and incubate the tissue with 5mL of prewarmed Digestion solution at 37°C under agitation (200rpm) conditions for 2hrs*.* For tissue storage, place minced tissue in freezing medium and when desired thaw the tissue in prewarmed Ad-DF basal medium, centrifuge the tube at 200g for 5 min, aspirate the supernatant and proceed with tissue digestion.

- - 1. Every 30 minutes of incubation, vortex the mixture for 10 seconds. !**CRITICAL STEP** Care should be taken to avoid the over-digestion of the material. Digestion is complete when microscopic examination shows clumps of cells free from the stroma. If the tissue is over-digested the cell viability will be extremely low.
    2. When digestion is complete ***(Figure 2)***, add 10mL of Transport medium to dilute collagenase, pass the digested material through 100-µm cell strainer and pellet the material by centrifuging at 200g for 5 minutes.
    3. Resuspend the pellet in 3mL of 4:1 mixture of ammonium chloride and cold modified HBSS for 3 minutes on ice to eliminate red blood cells. This step is not required for minced tissues that have been frozen.
    4. Add 10mL of Transport medium to the pellet material and centrifuge at 200g for 5 minutes. Discard the supernatant containing lysated red blood cells.
    5. Resuspend the pellet with 5mL of Ad-DF basal medium and centrifuge at 200g for 5minutes. Remove the supernatant and keep cells on ice.
  1. **Seeding of breast cells to generate organoids**

!**CRITICAL STEP** Matrigel is stored at -20°C and requires thawing on ice at 4°C for 2-3 hours. Freeze-thaws should be minimized by aliquotting into one time use aliquots. Matrigel will solidify at room temperature, so work quickly and keep the basement matrix cold through the process.

- - 1. By gently pipetting place 30μL drops of Matrigel on pre-warmed 24-well plates (1 drop/well), avoiding formation of air bubbles***.*** Incubate the plate 30 minutes at 37°C (until the matrix is solidified).
    2. By gently pipetting resuspend cell pellet in Matrigel and seed 20μL drops of Matrigel-cell suspension on pre-coated 24-well plates (1 drop/well) ***(Figure 3).*** Incubate the plate 1hr at 37°C (until the matrix is solidified).
    3. When the matrix is solidified, add 500µL of Human breast organoid medium.
    4. Incubate the material under standard tissue culture conditions (37°C, 5% CO2).
    5. Change media every 4 days.
    6. Tumor organoids should be visible within 2-3 days and ready for passaging around 1 week day of culture ***(Figure 4)***. Normal organoids grow slowly and are smaller than tumor one*.*
  1. **Organotypic culture maintenance**

Grow the organoids for 7 days. The general split ratio used for a 24 well is 1:2 depending on the number and size of the organoids. **!CRITICAL** For the following steps precoat all the tubes, the pipettes and tips with FBS to avoid attachment of the tissue to the plastic.

- - 1. Keep the plates on ice. Remove the medium from each 24 well, add 500µL of cold PBS to wash organoids. Aspirate PBS and add 500µL of cold PBS.
    2. Using p1000 pipette tips displace Matrigel from the wells and collect the material (PBS and organoids in Matrigel) in a 15mL tube and centrifuge it at 200g for 5 minutes at 4°C.
    3. Remove the supernatant and add Cell Recovery solution (~100μL each well). Incubate organoids in this solution for 1 hr at 4°C in order to gentle digest Matrigel and release cells with minimal cell damage.

Alternatively, 2,5 U/mL pre-warmed dispase (~100μL each well) can be used for 10 minutes at 37°C followed by addition of 10mL of cold PBS.

- - 1. Centrifuge organoids at 200g for 5 minutes at 4°C and aspirate supernatant.
    2. Add 5mL of cold PBS and centrifuge organoids at 200g for 5 minutes at 4°C to wash cells.
    3. Aspirate the supernatant completely. At this step, it is possible to freeze the organoids for storage or to keep organoids in culture. If organoids freezing is required, follow the procedure described in next paragraph.
    4. For organotypic culture expansion, further digestion can be performed using pre-warmed TrypLe solution (~100μL each well) for 5 minutes at 37°C followed by gentle pipetting (p1000). This step is not required if organoids are smaller than 50 μm.
    5. Add 5mL of Ad-DF basal medium and centrifuge 200g for 5 minutes.
    6. Remove supernatant and wash cells by adding 5mL of Ad-DF basal medium and centrifuge 200g for 5 minutes at 4°C.
    7. Remove supernatant and proceed with step 4.3.
  1. **Cryopreservation and thawing of organoids**

**!CRITICAL STEP** The freezing/thawing procedure is not efficient because of decreased viability of thawed organoids. For this reason, freeze as higher quantity of organoids as possible (8-12 well for each cryovial).

- - 1. **Cryopreservation of organoids**

Proceed as described in the paragraph **6.4** and then gently resuspend the organoids in 1mL of cold freezing medium (6.4.6). Transfer the suspension to cryovials and place them into ice bucket. Transfer the vials into a Cell Freezing Container at -80°C for 24 hrs, and then transfer them into liquid nitrogen vapor. **!CRITICAL STEP** The freezing medium contains DMSO, which is toxic to the cells at room temperature, work quickly and do not exceed a total of 5 min between adding the freezing medium to the organoids and transferring them to −80 °C.

- - 1. **Thawing of organoids**
       1. Pre-warm a water bath and 10mL of Ad-DF basal medium in a 15mL tube to 37°C.
       2. Incubate the cryovial in the 37°C water bath, stopping when the ice is almost completely thawed. Quickly transfer the cells to the pre-warmed 15-mL tube containing basal medium.
       3. Centrifuge the tube at 200g for 5 min and aspirate the supernatant.
       4. Seed organoids following the procedure described in the paragraph 4.3*.*

**5. Figures**


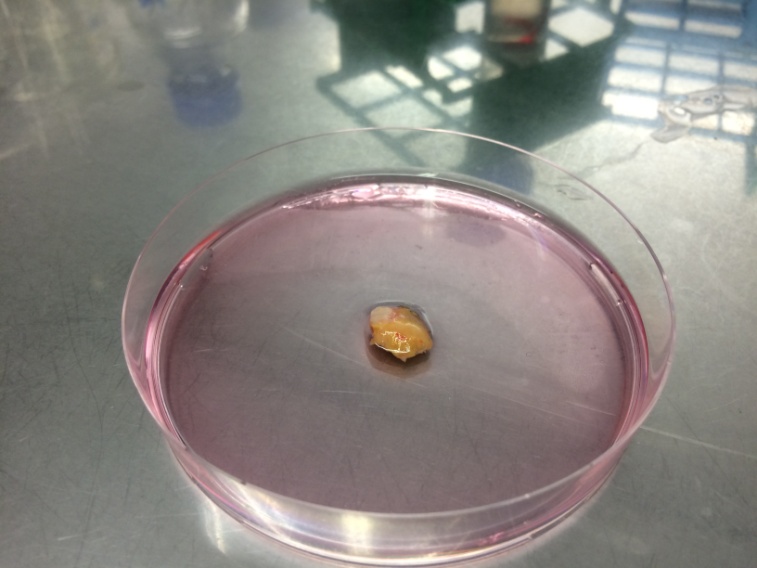

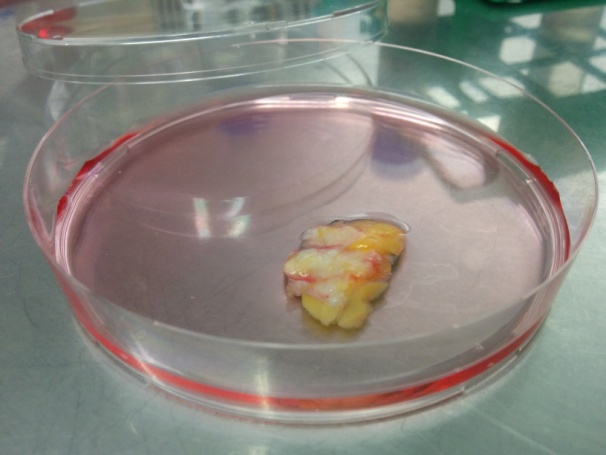


***Figure 1*.** Representative images of normal (left) and tumoral (right) biopsies of human breast tissue.


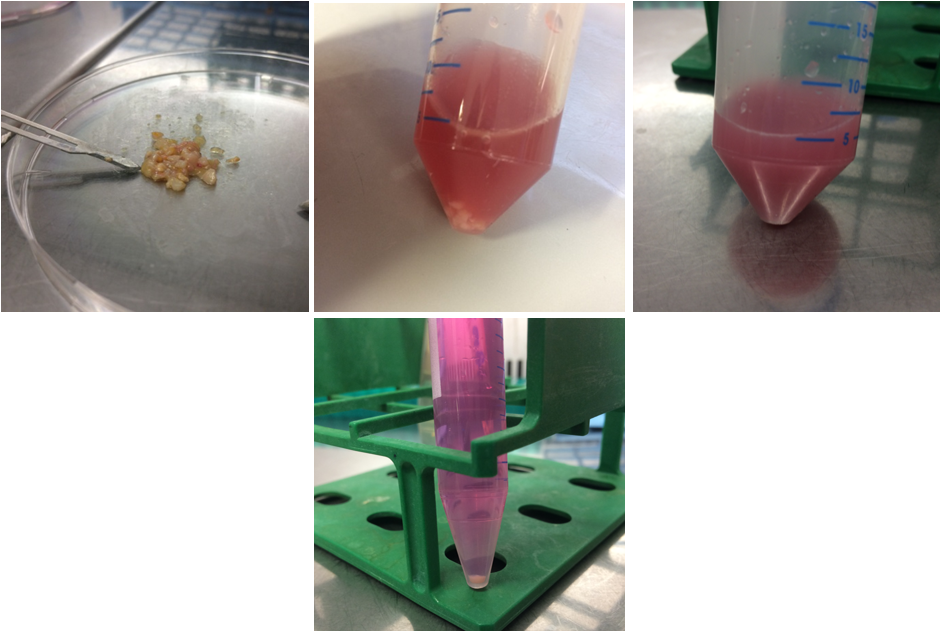


***Figure 2*.** Representative images of i) minced tumor tissue before (top, left and middle) and after 2 hours of incubation with Collagenase A (top, right), and ii) cells extracted from the tissue (bottom).


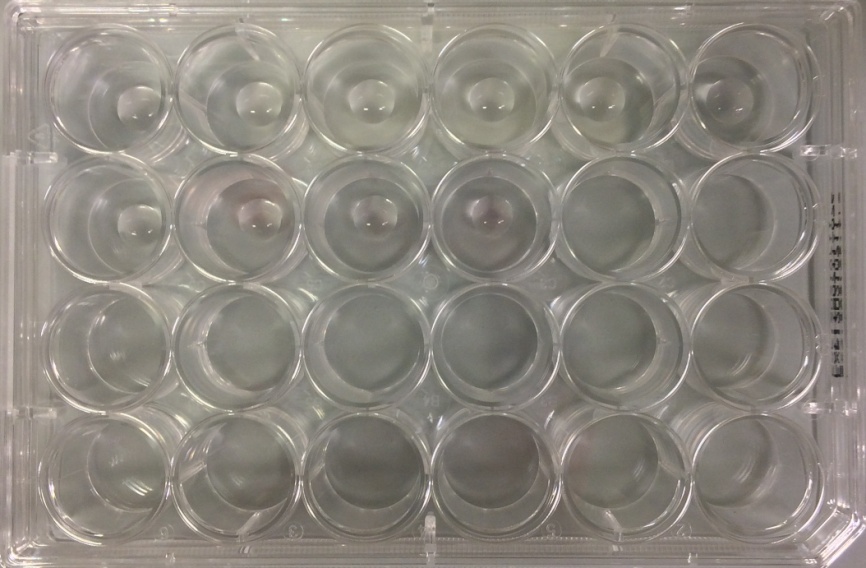


***Figure 3*.** Representative image of drops containing Matrigel-embedded tumor cells plated in the 24-wells plate.

**
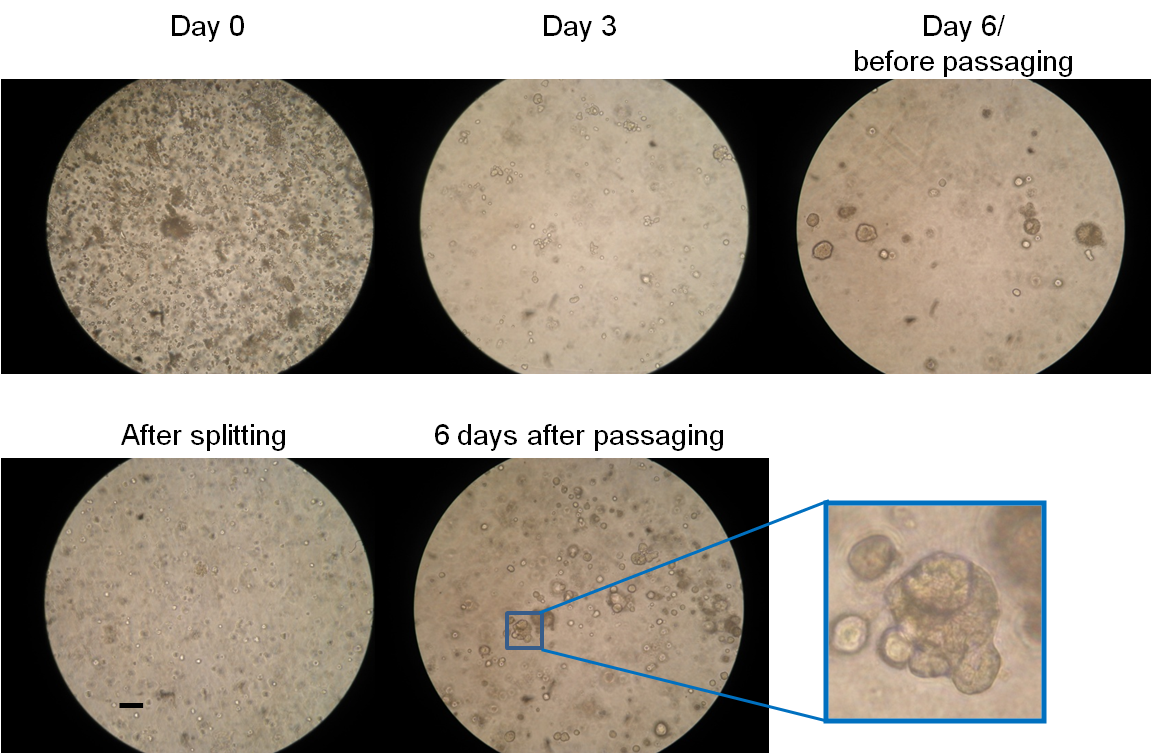
**

***Figure 4*.** Representative images of breast tumor organoids at different time points. Scale bar (100μm) is indicated.
